# Bacterial osmoprotectants—a way to survive in saline conditions and potential crop allies

**DOI:** 10.1093/femsre/fuaf020

**Published:** 2025-05-16

**Authors:** Aleksandra Goszcz, Karolina Furtak, Robert Stasiuk, Joanna Wójtowicz, Marcin Musiałowski, Michela Schiavon, Klaudia Dębiec-Andrzejewska

**Affiliations:** Faculty of Biology, Department of Geomicrobiology, Institute of Microbiology, University of Warsaw, Ilji Miecznikowa 1, 02096 Warsaw, Poland; Department of Microbiology, Institute of Soil Science and Plant Cultivation, State Research Institute, Czartoryskich 8, 24100 Puławy, Poland; Faculty of Biology, Department of Geomicrobiology, Institute of Microbiology, University of Warsaw, Ilji Miecznikowa 1, 02096 Warsaw, Poland; Faculty of Biology, Department of Plant Anatomy and Cytology, Institute of Experimental Plant Biology and Biotechnology, University of Warsaw, Ilji Miecznikowa 1, 02096 Warsaw, Poland; Faculty of Biology, Department of Geomicrobiology, Institute of Microbiology, University of Warsaw, Ilji Miecznikowa 1, 02096 Warsaw, Poland; Department of Agricultural, Forest and Food Sciences (DISAFA), University of Turin, Largo Paolo Braccini 2, 10095 Grugliasco, Italy; Faculty of Biology, Department of Geomicrobiology, Institute of Microbiology, University of Warsaw, Ilji Miecznikowa 1, 02096 Warsaw, Poland

**Keywords:** osmotic stress mitigation, overfertilization, biostimulation, plant growth-promoting bacteria, agrobiotechnology, soil regeneration, salinity management

## Abstract

Soil salinization, affecting 6.5% of arable land, deteriorates soil properties, reduces microbiota activity, hinders plant growth, and accelerates soil erosion. Excessive salt induces physiological drought and toxicity stress in plants, causing chlorosis, ion imbalances, and enzyme disruptions. This paper discusses microorganisms’ resistance mechanisms, plant responses to salt stress, and summarizes current knowledge on bacterial osmoprotectants and their functions. It also reviews emerging agrobiotechnological strategies using microbial osmoprotectants to remediate salinized soils and enhance plant growth and productivity under salt stress. Osmoprotectants stabilize proteins, buffer redox potential, and retain water, thus alleviating osmotic stress and promoting bacteria and plants growth. Their application improves soil properties by enhancing aggregate formation, water permeability, moisture content, cation exchange capacity, and ion availability. Despite extensive literature on the function of osmoprotectants, the knowledge about their role in soil environments and agrobiotechnology applications remains limited. This paper indicates proposed research perspectives, including discovering new osmoprotectants, their correlation with soil fertilization, interactions with the soil microbiome, and plant responses. It also identifies significant knowledge gaps in these areas, highlighting the need for further studies to consolidate existing data and assess the potential of this approach to enhance soil health and crop productivity in saline environments.

## Teaser

This review explores microbial osmoprotectants as innovative agrobiotechnological tools to combat soil salinization, emphasizing their potential to improve soil health, enhance plant resilience, and address critical challenges in sustainable agriculture under salt stress.

## Introduction

Soil salinization, a primary cause of osmotic stress in plants, is a major threat to both the environment and agriculture, severely hindering cropland productivity (Litalien and Zeeb 2020). It is defined as the accumulation of salts in soil to levels that inhibit plant growth and alter soil microbiological diversity (Haswell and Verslues 2015, Zhao et al 2020). According to the Food and Agriculture Organization of the United Nations (FAO), ~6.5% of the world arable soils are saline or sodic, translating to an estimated annual loss of US$27.3 billion in crop productivity (Qadir et al. 2014). More specifically, the global area of land affected by salinity includes 424 million hectares of topsoil (0–30 cm) and 833 million hectares of subsoil (30–100 cm) (Negacz et al. 2022). Many theoretical models based on environmental data predict further crop losses due to soil salinization in the future (Zia et al. 2021). Forecasts suggest that soil salinization will escalate in the upcoming years, potentially affecting more than 50% of croplands by 2050 (Zia et al. 2021). These catastrophic scenarios are fuelling the scientific community to tackle the problem, yet there remains a significant gap in understanding the processes within saline environments and developing effective remediation strategies (Litalien and Zeeb 2020, Otlewska et al. 2020, Haj-Amor et al. 2022).

Salinization in soil is typically estimated by measuring electric conductivity (EC), soil pH, and exchangeable sodium percentage (ESP) (Litalien and Zeeb 2020, Otlewska et al. 2020). Three types of salt-affected soils are distinguished: saline soils (pH <8.5, EC >4 dS/m, and ESP <15), sodic soils (pH >8.5, EC <4 dS/m, and ESP >15), and saline-sodic (pH >8.5, EC >4 dS/m, and ESP >15) (Litalien and Zeeb 2020). A spatial analysis revealed that the global area of saline soils with EC >4 dS/m is 16 646 000 km^2^ (Negacz et al. 2022). Natural salinization can occur due to the weathering of salty minerals or the influence of sea breezes; however, anthropogenic factors are primarily responsible for the current salinization crisis (Litalien and Zeeb 2020, Haj-Amor et al. 2022). Unsustainable agricultural practices, particularly improper irrigation and extensive fertilization, along with artificial flooding for aquaculture, are the main contributors of salt accumulation in croplands (Rütting et al. 2018, Xie et al. 2020, Singh 2021). Other human activities, including the utilization of road salts, oil extraction processes, and waste deposition industries like mining, paper pulp, steel, or cement production, further exacerbate soil salinity (Litalien and Zeeb 2020). Moreover, climate changes lead to reduced precipitation and high evaporation, accelerating soil salinization (Singh 2021, Haj-Amor et al. 2022), and rising sea levels cause saltwater intrusion in coastal and inland aquifers, worsening the adverse effects of salinity (Hu and Lindo-Atichati 2019).

Salt ions, such as Na^+^, K^+^, Cl^−^, NH_4_^+^, Mg^2+^, Ca^2+^, and SO_4_^2−^, introduced into the soil in excess through natural and anthropogenic activities, negatively affect the plant’s growth, limiting cropland productivity (Rütting et al. 2018, Litalien and Zeeb 2020, Pantha et al. 2022). Soil salinities ranging from 2 to 4 dS/m can adversely affect yields of sensitive crops, while salinity levels above 8 dS/m generally cause a significant reduction in growth of most crops and plants (Hassani et al. 2021). The extensive presence of salt ions in soil alters its physical structure, by interacting with soil particles, reducing soil porosity, and limiting root access to oxygen and water (Litalien and Zeeb 2020).

Consequently, salinity primarily impacts plants by reducing their ability to uptake water from the soil due to an osmotic gradient, where root cells exhibit a higher osmotic potential than the surrounding saline environment (Kido et al. 2019). This water deficit causes protein misfolding, loss of turgor pressure, and accumulation of reactive oxygen species (ROS) and reactive nitrogen species (RNS) (Angon et al. 2022, Saddhe et al. 2019, Sunita et al. 2020). High soil salinization also affects the soil microbial quality, reducing the growth and activity of microorganisms, which contributes to soil organic matter depletion (Haj-Amor et al. 2022).

Biological methods for saline soil regeneration are increasingly emerging as a valuable and low-cost alternative for mitigating osmotic and ion toxicity stress in saline agricultural soils (Kumar Arora et al. 2020, Orhan 2021). In this context, halotolerant and halophilic microorganisms like archea, fungi, or bacteria are particularly noteworthy, as they can grow in the presence of a wide range of environmental salinities (Etesami and Beattie 2018, Kumar Arora et al. 2020, Zhao et al. 2022). These salt-adapted microorganisms not only survive in saline soils, but also offer various benefits to the soil ecosystem (Etesami and Beattie 2018, Kumar Arora et al. 2020, Naitam et al. 2023 Orhan 2021, Zhang et al. 2024).

Given the diversity of halotolerant microorganisms, this study places particular emphasis on halotolerant and halophilic bacteria (HHB), owing to their unique metabolic traits and high potential for agricultural applications. HHB achieve tolerance for high salt concentrations by various mechanisms, including removal of intracellular salt via membrane transport proteins, exopolysaccharide (EPS) production, salt-in salt-out strategies, or osmoprotectants biosynthesis and uptake (Sleator and Hill 2002, Vauclare et al. 2015, Orhan 2021). Additionally, many HHB exhibit plant growth-promoting properties. Due to their intrinsic metabolic and genetic adaptations, bioaugmentation with these bacteria and/or their metabolites could significantly alleviate osmotic and ion toxicity stress in agricultural soils (Etesami and Beattie 2018).

Among the secondary metabolites produced by HHB, osmoprotectants hold great potential for regenerating saline agricultural soils (Sleator and Hill 2002, Kumar Arora et al. 2020). Osmoprotectants are molecules characterized by high solubility, no net charge at physiological pH, and lack of interactions with proteins or other biomolecules (Slama et al. 2015, Omara et al. 2020). Because of their compatibility with cellular functions, they are often referred to as “compatible solutes.” Osmoprotectants can accumulate within cells to high intracellular concentrations, without disrupting crucial cellular processes, like DNA processing or enzymatic activity (Sleator and Hill 2002). Their primary biological role is to maintain intracellular osmotic balance and stabilize protein under conditions of salinity or temperature stress, preventing cell dehydration (Ziegler et al. 2010).

Recent discoveries in the field of agrobiotechnology have shown that bacterial osmoprotectants can improve the microbiological and physical quality of agricultural soils, and increase plant resilience in response to abiotic stresses (Fouda et al. 2021). Microbes take up osmoprotectants from the extracellular environment through membrane transport proteins, whose expression is triggered by osmotic pressure (Sleator and Hill 2002). One of the best-studied examples is a BCCT (betaine–choline–carnitine-transporter) family of carriers, ubiquitous in many microorganisms (Ziegler et al. 2010). Since the uptake of compatible solutes requires less energy than their biosynthesis, the addition of osmoprotectants to scarce soil resources could improve salinity tolerance while also stimulating the growth of the entire microbiome (Sleator and Hill 2002, Ziegler et al. 2010). Osmoprotectants could directly influence the water structure by strengthening the water–water bonds (Sleator and Hill 2002, Dashnau et al. 2005, Guo and Friedman 2009). They could also indirectly improve water retention in the soil through the stabilization of soil biofilms (Panuszko et al. 2016), which, in turn, promotes soil aggregation (Guo et al. 2018). Regarding plants, external application of osmoprotectants to saline agricultural soil can stimulate their biomass, increase photosynthetic activity and reduce both ROS and RNS production (Mäkelä et al. 1999, Semida et al. 2020, Vurukonda et al. 2016). Moreover, seed priming with osmoprotectants can improve germination, seed viability and plant tolerance to salinity, thereby supporting overall plant growth (Ambreen et al. 2021).

**Figure 1.**
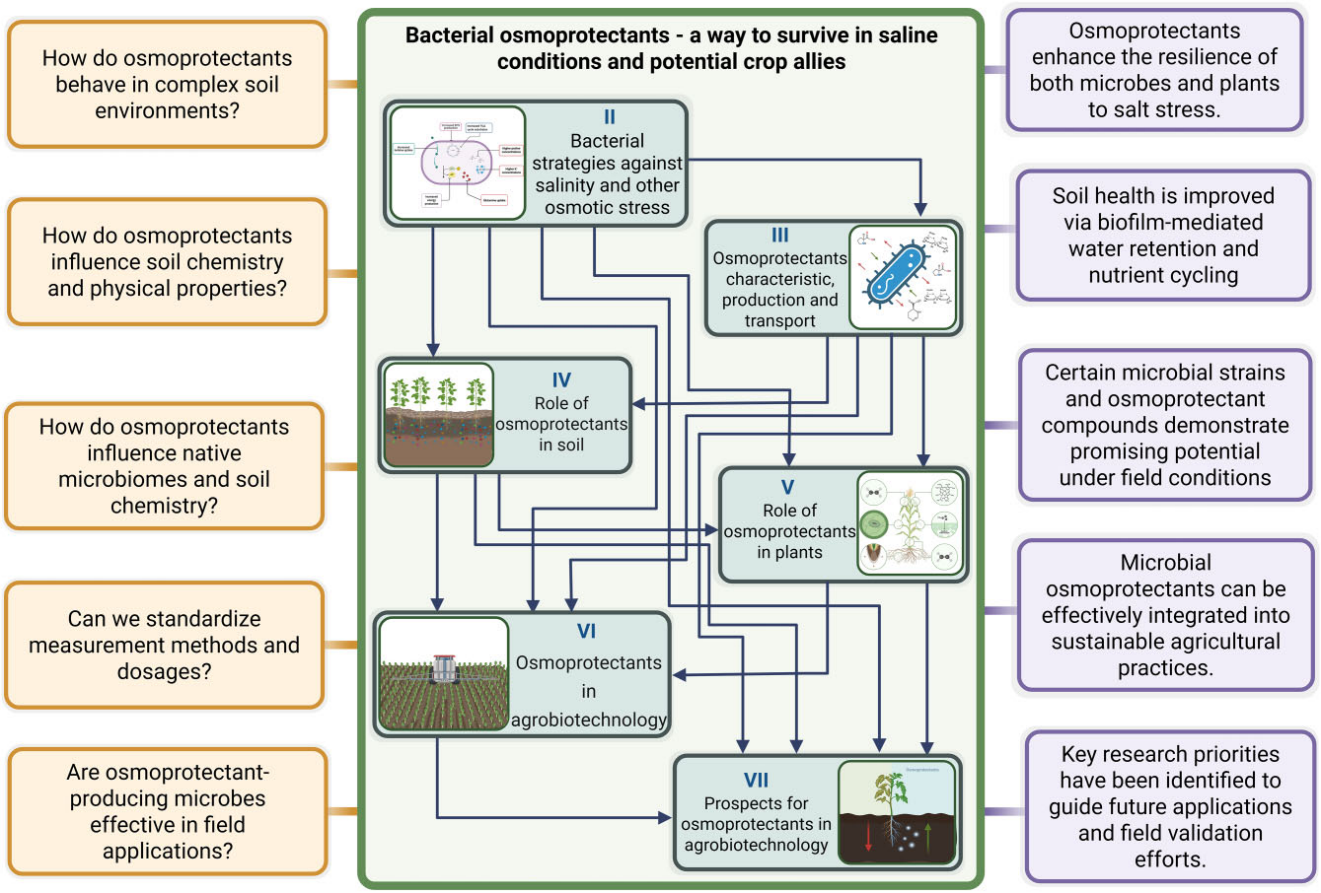
Schematic representation of the interconnections between the key components of the review (central box), illustrating how they integrate fundamental research questions on bacterial osmoprotectants with their potential applications in agriculture (boxes on the left), and highlighting the principal outcomes of the review (boxes on the right).

The adaptive strategies of bacteria to cope with osmotic stress conditions, particularly due to salinization, can provide guidance and support for assisting plants under increasingly challenging environmental conditions. The processes used by microorganisms can be exploited in agrobiotechnology to mitigate the effects of salinization and other causes of osmotic stress occurring in soil, thereby protecting crops. In the light of this, this review addresses the ability of bacteria to adapt to the varying environmental osmolarity and the role of osmoprotectants in saline soil regeneration and the enhancement of crop resilience to high salinity. The last part of this paper indicates directions for future research aimed at gaining a better understanding of the role of osmoprotectants in soil and their potential application in agrobiotechnology. This review highlights critical knowledge gaps and emerging research questions, offering a synthesis of current findings and proposing new perspectives to guide future studies on the use of osmoprotectants in agrobiotechnology (Fig. 1)

## Bacterial strategies against salinity and other osmotic stress

**Figure 2.**
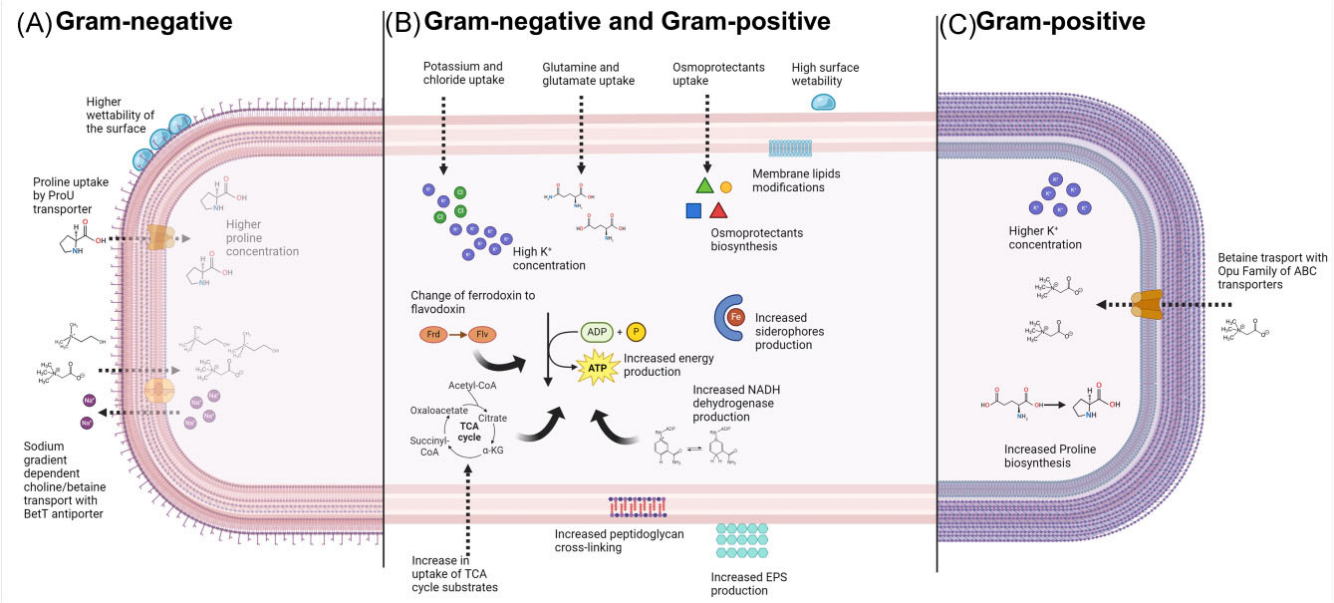
Bacterial strategies against salinity and osmotic stress. (A) Mechanisms predominant in Gram-negative bacteria. (B) Common mechanisms in both Gram-positive and Gram-negative bacteria. (C) Mechanisms more developed in Gram-positive bacteria.

Bacterial survival across diverse habitats depends on their ability to adapt to osmotic changes in their environment. Osmotic stress arises from solute imbalance between the bacterial cytoplasm and the external environment, causing water movement across the cell membrane that can potentially lead to bacterial swelling and rupture in hypertonic conditions or shrinking and dehydration in hypotonic ones (Romantsov and Wood 2016). These osmotic imbalances can affect vital cellular components and processes by disrupting proteins functionality (Nagavi-Alhoseiny et al. 2019, Sánchez et al. 2004, White et al. 2022), metabolic pathways (Sánchez et al. 2004), RNA polymerase–DNA interactions (Li et al. 2021), and other crucial cellular activities like DNA replication and protein synthesis (Nagavi-Alhoseiny et al. 2019). To maintain cellular homeostasis, bacteria have evolved adaptive mechanisms (Fig. 2) that vary depending on osmotic pressure levels or species-specific traits (Godard et al. 2020).

### Changes in general bacterial metabolism

In response to prolonged salinity exposure, bacteria adjust at multiple levels of biological processes, including gene expression, regulation, and cellular metabolism (Kloska et al. 2022). These changes in metabolic pathways typically take more time than other adaptation strategies, such as alterations in cell wall content, but can effectively reduce the stress associated with long-term exposure. They vary from minor adjustments focusing on a single substrate to major alterations in central metabolism and energy acquiring processes (Kloska et al. 2022). Major changes are often caused by ROS and fluctuations in available compounds, especially organic carbon sources (Xing et al. 2023). Godard et al. (2020) observed that *Bacillus megaterium* exhibited enhanced expression and concentration of proteins involved in oxidative stress response and iron metabolism when exposed to 0.6 M NaCl concentration. This response included increased levels of the PerR regulator, NADH dehydrogenase, iron-binding proteins, and various cytochromes. Furthermore, flavodoxins, which replaced ferredoxins under stress, showed up to 6.6-fold increase in concentration, while genes related to Fe–S cluster repair were upregulated ~2-fold. Iron acquisition-related proteins also saw dramatic increases in both expression (up to 15-fold) and protein concentration (up to 12-fold), indicating an adaptation mechanism involving iron scavenging under high salinity. This response is partially explained by the decreased solubility of oxygen at high salt concentrations, which affects Fe metabolism by limiting the bioavailability of Fe required for key redox processes.

As oxygen levels drop in saline soils, microbial communities shift their metabolism (Bok et al. 2023). Many soil and aquatic bacteria are facultative aerobes capable of switching from aerobic respiration to microaerophilic or anaerobic pathways when oxygen becomes scarce (Zumft 1997). Under these conditions, processes such as denitrification are often activated, with bacteria utilizing oxidized nitrogen species (e.g. nitrate and nitrite) as alternative electron acceptors (Zumft 1997, Shapovalova et al. 2008). Salt-induced oxygen depletion upregulates denitrification genes, enabling energy conservation through nitrate and nitrite reduction. Halotolerant and haloalkaliphilic bacteria such as *Halomonas campisalis* and *H. mongoliensis* exemplify this metabolic flexibility, as they can perform complete denitrification under moderate salinity (Shapovalova et al. 2008). Similarly, *Bacillus halodenitrificans*, a halophilic strain isolated from a solar saltern, grows anaerobically via nitrate and nitrite respiration even at NaCl concentrations up to 4.25 M (≈25% salt) (Denariaz et al. 1989). Denitrification driven by salt-induced hypoxia significantly impacts soil nitrogen dynamics by converting bioavailable nitrate into gaseous N₂O and N₂ (Yang et al. 2022), thereby depleting soil nitrate reserves and potentially leading to transient nitrite accumulation, which can be toxic to plants and soil organisms (Chamandoost et al. 2016).

Additional changes in microbial metabolism are evident in other pathways, like carbohydrate, amino acid fatty acid metabolism, as well as leucine degradation. These adjustments lead to the production of metabolites like methyl-butanal and 3-methyl-butanol, which may be specific to salt stress (Park et al. 2022). Under saline stress, bacteria often tend to increase their energy production, for example, by substrate-level phosphorylation (Pta–Ack pathway) and the anaplerotic function of the tricarboxylic acid cycle (TCA) (Arense et al. 2010) (Fig. 2B). Variations in the accumulation of substrates in the TCA cycle seems to be common across different bacterial taxa, including *Sphingomonadaceae, Pyrinomonadaceae, Nitriliruptoraceae, Bacillaceae, Halomonadaceae*, and *Pseudomonadaceae* (Wang et al. 2021a). Subsequently, upregulation of TCA cycle results in higher secretion of these metabolites, such as fumaric acid, malic acid, and oxalic acid.

### Mechanosensitive channels

Mechanosensitive (MS) channels are integral components of the bacterial plasma membrane playing a pivotal role in sensing and responding to mechanical stresses, including osmotic changes. The MS channel of large conductance (MScL) and the MS channel of small conductance (MScS) are among the most studied MS channels in bacteria, particularly in *Escherichia coli* (Cox et al. 2018). MS channels facilitate the rapid passage of ions and small molecules upon activation, thereby preventing potential damage from hypoosmotic stress (Wood 2007, Booth 2014). For example, in *Paraburkholderia graminis*, a rhizosphere-associated bacterium, MScL-like and MScS-like channels are vital for survival during osmotic shifts such as those caused by rainstorms (Miller et al. 2020).

The gating of MS channels, transitioning from a closed to an open state, occurs in response to increased tension within the lipid bilayer of the plasma membrane (Booth 2014), which primarily results from transmembrane osmotic gradients. In hypoosmotic environments where the external osmolarity is significantly lower than the internal cytoplasmic concentration, water influx increases the cell’s internal pressure (Kung 2005, Booth 2014).

### Changes in cell membrane and wall

Osmotic stress induces significant changes in bacterial cell membranes and walls. Research indicates that exposure to osmotic stress can lead to alterations in cell surface hydrophobicity (CSH) (Karagulyan 2022), adjustments in membrane permeability and outer membrane porin expression (Bremer and Krämer 2019), and increased peptidoglycan cross-linking affecting resistance to osmotic stress (Karagulyan et al. 2022). For example, CSH often tends to increase, enhancing the ability of bacteria to adhere to surfaces, and potentially decreasing their area of exposure to high osmotic conditions by binding to neutral surfaces. The increase of CSH in response to osmotic stress is generally more pronounced in Gram-negative (G−) bacteria compared to Gram-positive (G+) (Karagulyan 2022) (Fig. 2A). From an environmental perspective, the CSH parameter of the bacterial cell walls is associated with increased water retention of soil colonized by bacteria. It has been noted that soils with prevailing G+ bacteria content possess higher wettability properties than those dominated by G− bacteria (Karagulyan et al. 2022) (Fig. 2A). This phenomenon is likely due to an increase in proteins and hydrocarbon concentration within the cell wall, accompanied by a decrease in polysaccharides content (van der Mei et al. 2003).

Bacterial surface hydrophobicity can be expressed by the contact angle between the surface and the water coating it. Overall, osmotic stress and hyperosmotic conditions increase the hydrophobicity of the bacterial surface, though this effect varies depending on growth conditions. Under submerged growth bacterial hydrophobicity generally increases, but the response can be different for surface growth. Depending on whether bacteria are G+ or G−, the contact angle can rise or remain stable under elevated salinity. In G+ bacteria, such as *Bacillus subtilis*, the parameter of CSH tends to remain steady. In contrast, G− bacteria like *Pseudomonas fluorescens* show a similar increase in surface hydrophobicity similarly as in submerged growth. Additionally, higher salinity can lead to an increase in zeta potential t, which can contribute to a more robust and stable cell wall (Karagulyan et al. 2022).

The composition and physiological parameters of cell membranes undergo various changes, such as alterations in the structure and make-up of phospholipids (Chwastek et al. 2020). Chwastek et al. (2020) found that that the bacterium *Methylobacterium extroquens* significantly alters its lipid membrane fraction, affecting 90% of its lipid types with phosphatidylcholine showing major acyl chain chain modifications (Chwastek et al. 2020). Modifying membrane lipids composition is another adaptation to salinity. For example, anionic phospholipids such as phosphatidylglycerol and cardiolipin increase, facilitating osmosensory transporters and MS channels, which regulate solute fluxes (Romantsov and Wood 2016).

### Production of EPS

Another mechanism by which bacteria evolved to survive under salinized conditions is the production of polymeric EPS. EPS are synthesized from a diverse array of precursors, including glucose, trehalose, rhamnose, and fructose, among others, which are polymerized through enzymatic pathways involving glycosyltransferases (Balducci et al. 2023). In addition to these sugars, EPS can include uronic acids, amino sugars, and occasionally noncarbohydrate substituents, which contribute to the physicochemical properties of the EPS and their function in biofilm formation (Donlan 2002). The composition of EPS is highly variable depending on bacterial species or environmental conditions, such as soil salinity. Under such conditions, the composition of bacterial biofilms tilts toward higher concentrations of sugars and functional groups such as carboxyl, hydroxyl, and phosphate (Liu et al. 2023b). This preference can be described by the affinity of these groups for chelating Na^+^ ions, which reduces their uptake through bacterial membranes (Liu et al. 2023a). In adapting to high salinity, the EPS matrix binds cations such as Na^+^ ions, thus significantly lowering their effective concentration near the cell surface and, consequently, the osmotic pressure on the bacterial membrane. It also retains water, which helps in reducing the osmotic gradient (Padgett-Pagliai et al. 2022). Biofilms, structured communities of microorganisms embedded within the EPS matrix, offer enhanced protection against environmental stressors, facilitating microbial colonization and survival in harsh habitats (Padgett-Pagliai et al. 2022). Interestingly, the bacterial biofilm surface is covered by a hydrophobic and redox-sensitive surface layer called BslA (Hobley et al. 2013), but water and nutrients still flux into biofilm through osmotic gradient forces established by high amounts of secreted exopolymers (Yan et al. 2017). However, as the biofilm expands, cells within it experience increased osmotic stress. To prevent this, bacteria strains naturally producing higher amounts of biofilm also synthesize compatible solutes to alleviate this stress (Yan et al. 2017).

### Rapid uptake of potassium

The rapid uptake of K^+^ ions is a critical response mechanism in bacteria to counteract osmotic stress caused by hypersaline conditions. This uptake is facilitated by specialized K^+^ transporters, such as Kup/HAK/KT and Trk systems (Epstein 2003). Sensor kinases such as KdpD play a primary role in this process as they detect decreases in intracellular K^+^ concentration and trigger the activation of the Kdp-ATPase system (Heermann and Jung 2010). The resulting increase in internal K^+^ concentration serves as an initial adjustment to the cell’s internal osmotic pressure, thereby equalizing the osmotic pressure across the plasma membrane and countering the external osmotic forces (Heermann and Jung 2010). For example, in *Sinorhizobium meliloti*, the Trk system is primarily responsible for the accumulation of K^+^ following an osmotic upshift, making it essential for the bacteria growth under hyperosmotic conditions (Domínguez-Ferreras et al. 2009) (Fig. 2). Similarly, in *E. coli* a substantial increase in intracellular osmolality has been observed as part of its osmotic stress response, which includes a rapid uptake and possible efflux of K^+^ depending on the severity of the stress and the type of osmolytes involved (Shabala et al. 2009). Under mild osmotic stress, the intracellular concentration of K^+^ ions increases, which is accompanied by a corresponding increase in glutamate concentration (Godard et al. 2020). However, under more severe osmotic conditions, *E. coli* shifts its strategy replacing glutamate, which was previously preferred, with proline. As a result, as proline concentration rapidly increases, glutamate concentration concurrently decreases (Godard et al. 2020). This shift may be caused by higher, cytotoxic, K^+^ concentrations in cells. The effectiveness of K^+^ uptake and subsequent osmotic stress response can be significantly influenced by other ions, such as Na^+^, which may lead to competitive interactions affecting K^+^ homeostasis and the overall cellular response to osmotic changes. Conditions like low pH can markedly increase K^+^ uptake (Ochrombel et al. 2011). The concentration and flux of K^+^ differ between G+ and G− bacteria. Gram-positive bacteria naturally maintain high concentration of K^+^ions (even over 0.1 M) under standard growth conditions (Bremer and Krämer 2019), likely because they require bigger internal pressure to stretch the much thicker peptidoglycan sacculus when cells double their volume before dividing (Foo et al. 2015, Erickson 2017). These interactions highlight the complexity of the osmotic stress response in bacteria, involving a finely tuned balance between ion uptake, intracellular adjustments, and external environmental conditions.

### Salt-in and salt-out strategies

In response to osmotic stress two main strategies are commonly employed: the “salt-in” strategy, where bacteria increase in concentration of intracellular salts to align with extracellular concentrations, and a second strategy “salt-out” that alleviates salt stress in the periplasm by synthesizing or taking up compatible solutes (Hernández-Canseco et al. 2022).

The first strategy involves accumulating K^+^ and chloride (Cl^−^) ions within cells to match external concentrations, requiring significant enzymatic adjustments, to ensure protein in high salt conditions (Lanyi 1974) Moreover, the proteome of the organisms using this strategy is predominantly acidic, adapted to function in high-salt environments, and their proteins typically lose stability or denature in low-salt environments. Hence, these organisms are generally unable to survive in hypotonic environments (Oren 2008). Although this strategy is less energetically costly than the second, it is employed by relatively few microorganisms, mostly those from the archaea domain, like members of the phylogenetic family *Haloanaerobiales* (Oren 2008).

The “salt-out” strategy is more effective, but more energy-consuming. In this case, bacteria reduce cytoplasmic ion and salt concentration by accumulating compatible solutes, thereby alleviating osmotic stress. These solutes include proteins, sugars, carbohydrates, and alcohols, obtained through *de novo* synthesis, catabolism or acquisition from the environment via specific membrane transporters. In addition to these primary strategies, bacteria use MS channels, membrane and cell wall modifications, metabolic adjustments, production of EPS, and the uptake of potassium to cope with osmotic stress.

## Osmoprotectants characteristic, production, and transport

Osmoprotectants can be produced by microorganisms such as bacteria and archaea, as well as by plants and algae (Rathinasabapathi 2000). Among bacteria, the best known for osmoprotectant production are halophilic and halotolerant species, which synthesize ectoine and glycine–betaine (GB), among others (Tanimura et al. 2013). The existence of various synthesis pathways of osmoprotectants and the high diversity of these compounds enable bacteria to adapt and survive in a wide range of saline environments. Osmoprotectants and their characteristics are detailed below.

### Chemical characteristic

A wide variety of organic compounds belonging to many chemical classes serve as osmoprotectants in bacterial cells. They can be categorized into the following chemical classes: (i) amine, amino acids, and derivatives, (ii) quaternary ammonium, (iii) tertiary sulphonium, (iv) sugars, and (v) polyhydric alcohols (Table 1). These compounds can stabilize various structures in the cell, scavenge RNS and ROS, form a protective layer surrounding cellular structures and stimulate the cell to respond more efficiently to osmotic stress (Table 1). A detailed characterization of each of the different classes of osmoprotectants was provided below.

**Table 1.** Properties of osmoprotectants from various chemical groups, which confer to their osmoprotective capabilities.

Chemical class	Osmoprotective properties	References
Amine, amino acids, and deriatives	● Stabilize membranes and proteins ● Buffer cellular redox potential ● Scavenge ROS ● Influence the expression of genes related to osmoprotection ● Provide buffer capacities ● Bind water (zwitterions) ● Balance osmotic pressure	Kempf and Bremer (1998a), Sleator and Hill (2002), Wood (2007), Campbell and Farrell (), Takagi et al. (2016)
Quaternary ammonium	● Stabilize enzymes and proteins against denaturation caused by osmotic stress. ● Buffer cellular redox potential ● Balance osmotic pressure within cells. ● Provide buffer capacities ● By binding to different groups (such as methyl groups), increase their water solubility—increase solute amounts in cell	Slama et al. (2015)
Tertiary sulphonium	● Stabilize enzymes and proteins against denaturation caused by osmotic stress. ● Buffer redox activity ● Accumulate in high concentration without cytotoxicity effect ● Maintain the integrity of cellular membranes by interacting with lipid components, preventing destabilization under variable external osmotic conditions	Jawahar et al. (2019), Rajasheker et al. (2019)
Sugars	● Stabilize proteins by forming hydrogen bonds ● Serve as extracellular barrier around bacteria—EPS layer ● Form an EPS layer to protect bacterial cells	Rathinasabapathi (2000)
Polyhydric alcohol	● Stabilize membrane lipids and proteins at low water activity ● Protect cells from oxidative damage ● Balance osmotic pressure within cells ● Facilitate osmotic adjustment by managing water flux across the plasma membrane ● Negatively charged carbohydrates and sugar alcohols provide counterions for salt cations, e.g.K^+^	Shen et al. (1999), Wisselink et al. (2002), Roberts (2004)

#### Amine, amino acids, and derivatives

Amino acids are derivatives of hydrocarbons consisting of a central carbon atom covalently bonded to an amine group (-NH), a carboxyl group (-COOH), a hydrogen atom, and a specific side chain (Campbell and Farrell ). Amino acids are usually well soluble in water and can behave as zwitterions in solution, meaning they carry both positive (from the amino group) and negative (from the carboxyl group) ionic charges. Depending on the functional group in the side chain, amino acids can be acidic (e.g. glutamic acid), basic (e.g. lysine), or neutral (glycine, alanine, and leucine) (Campbell and Farrell ).

Due to their unique physicochemical properties, many microorganisms use amino acids and their derivatives as osmoprotectants in response to osmotic stress (Table 1) by either take up from the environment or *de novo* synthesis (Tables 2 and 3) to counteract water loss and maintain osmotic homeostasis (Kempf and Bremer 1998a, Sleator and Hill 2002, Wood 2007). By increasing osmotic pressure within the cell, amino acids and their derivatives can help maintain the integrity of cell membranes and protein structures and contribute to buffer the cellular redox potential, reduce RNS and ROS levels, and increase overall cell survival (Kempf and Bremer 1998a, Sleator and Hill 2002, Wood 2007, Takagi et al. 2016, Lushchak and Lushchak 2021) (Table 1).

**Table 2.** Production of osmoprotectants by bacteria.

Osmoprotectant	Microorganism producing	Substrate	Concentration	References
Amine, amino acids, and their derivatives				
Proline	*Bacillus licheniformis* NJ04	LB nutrient broth	32.31 mg/g	James and Umesh ()
	*B. subtilis*	OpuE	∼60 mg/l	Hoffmann et al. (2012)
	*Halobacillus halophilus*	Salt medium (G10) containing 1% glucose	6.18 μM/mg of protein	Saum and Müller (2007)
	*Pseudoalteromonas phenolica* KCTC 3208	Marine broth	∼2500 μM	Song et al. (2020)
Alanine	*B. subtilis* JH642 and DRB30	Spizizen’s minimal medium	239 and 207 mM	Zaprasis et al. (2015)
	*Desulfovibrio vulgaris* Hildenboroug	Lactate sulfate medium	17.53 µM/mg dry weight	He et al. (2010b)
	*Rhizobium* strains RlF16 and Rch60	Mannitol–aspartate–salts medium	95 and 146 nM/mg of proteins	Bouhmouch et al. (2001)
Arginine	*B. subtilis* DRB 17	Spizizen’s minimal medium	2.5 mM	Zaprasis et al. (2015)
Pipercolic acid	*Brevibacterium ammoniagenes* ATCC 6872	M63 minimal medium	130 µM/mg dry weight	Gouesbet et al. (1992)
	*Corynebacterium glutamicum*	Glucose-minimal medium	30 μg/g CDW h	Pérez-García et al. (2019)
Citruline	*B. subtilis* DRB17	Spizizen’s minimal medium	237 mM	Zaprasis et al. (2015)
Glutamate	*Arthrobacter globiformis*	Czapek medium	25.58 mg/g cells	Komarova et al. (2002)
	*Desulfovibrio vulgaris* Hildenboroug	Lactate sulfate medium	82.82 µM/mg dry weight	He et al. (2010b)
	*Rhodococcus erythropolis* E-15	Czapek medium	35.14 mg/g cells	Komarova et al. (2002)
Asparagine	*Arthrobacter globiformis*	Czapek medium	1.22 mg/g cells	Komarova et al. (2002)
	*Rhodococcus erythropolis* E-15	Czapek medium	1.90 mg/g cells	Komarova et al. (2002)
Carnitine	*E. coli* 044 K74	Minimal medium	0.5 M	Cánovas et al. (2007)
Quaternary ammonium				
Hydroxy proline	*B. subtilis*	Marine broth	2.9 µM/g—dry weight	Kim et al. (2017)
	*Halobacillus halophilis*	Marine broth	188.7 µM/g—dry weight	
	*Pseudomonas stutzeri*	Marine broth	0.8 µM/g—dry weight	
	*Virgbacillus pantothenicus*	Marine broth	27.2 µM/g—dry weight	
Glycine betaine	*Synechocystis* sp. PCC 6803	BG11 medium	64.29 µM/g—dry weight (1.89 µM/mg protein)	Ferreira et al. (2021)
β-alanine betaine	*B. subtilis* PanDE56S	Glucose	236.15 mg/l culture	Perchat et al. (2022)
Tertiary sulphonium				
DMSP (dimethylsulfoniopropionate)	*Gynuella sunshinyii* YC6258	MBM medium, MTHB	177.42 ± 3.2 pmol/μg protein/h	Wang et al. (2024)
Sugar				
Sucrose	*Synechococcus elongatus* UTEX 2973	BG11 medium	1.9 g/l day	Streeter (2003), Hagemann (2011)
Trehalose	*Brevibacterium* sp. SY361	Glucose, polypeptone, KH2PO4, MgSO4 × 7 H2O	12.2 mg/ml	Wang et al. (2008)
	*E. coli*	M9	1.7 g/l	Li et al. (2012)
Fructants				
Mannitol	*Lactobacillus brevis* 3-A5	Jerusalem artichoke extract	176.50 g/l	Cao et al. (2018)

**Table 3.** Bacterial membrane transporters responsible for the transport of osmoprotectants.

Transport system	Osmoprotectants	Microorganism	References
EctP	Betaine, ectoine, and proline	*Corynebacterium glutamicum*	Wood et al. (2001)
BetP	Betaine and glycine	*Corynebacterium glutamicum*	Wood et al. (2001)
BetT	Betaine, carnitine, and choline	*Haemophilus influenzae, Pseudomonas syringae*	Chen and Beattie (2008)
BetU	Betaine, carnitine, and choline	*E. coli*	Ly et al. (2004)
BetS	Glycine betaine/proline	*S. meliloti*	Boscari et al. (2002)
BCCT	Betaine, carnitine, and choline	*Vibrio parahaemolyticus*	Ongagna-Yhombi et al. (2015)
BusA and QacT	Dimethylsulfoniopropionate, carnitine, and proline	*Pediococcus pentosaceus, Tetragenococcus halophila*	Baliarda et al. (2003)
Cbc	Betaine, carnitine, and choline	*Pseudomonas aeruginosa PA14*	Chen et al. (2010)
GabP	Proline	*B. subtilis*	Zaprasis et al. (2014)
GltT	Glutamate	*B. subtilis*	Zhu and Stülke (2018)
GgtB	Glucosylglycerol, sucrose, and trehalose	*Synechocystis sp. PCC6803*	Mikkat and Hagemann (2000)
OpuA	Dimethyl sulfoacetate, GB, homobetaine, and proline–betaine	*Bacillsus subtilis, Lactococcus lactis*	Wood et al. (2001), Hoffmann and Bremer (2011), Hoffmann and Bremer (2017)
OpuB	Choline	*Bacillsus subtilis*	Jebbar et al. (1997), Hoffmann and Bremer (2011), Hoffmann and Bremer (2017)
OpuC	Carnitine, choline, choline-o-sulfate, crotonobetaine, dimethyl sulfoacetate, ectoine, gamma-butyrobetaine, GB, homobetaine, and proline–betaine	*Bacillsus subtilis, Pseudomonas syringae*	Jebbar et al. (1997), Chen and Beattie (2007), Hoffmann and Bremer (2011), Hoffmann and Bremer (2017)
OpuD	Dimethyl sulfoacetate, GB, and proline	*Bacillsus subtilis*	Wood (2007), Hahne et al. (2010), Hoffmann and Bremer (2017)
OpuE	Proline	*Bacillsus subtilis*	Hoffmann and Bremer (2011), Hoffmann and Bremer (2017)
Porter II	Carnitine and GB	*Listeria monocyogenes*	Wood et al. (2001)
Prop and ProU	Glycine betaine, pipecolic acid, and proline–betaine	*E. coli, Corynebacterium glutamicum*	Gouesbet et al. (1992), Haardt et al. (1995), Wood et al. (2001)
PutPA	Proline	*E. coli*	Wood (2007)
TeaABC	Ectoine and hydroxyectoine	*Halomonas elongata*	Tetsch and Kunte (2002), Kuhlmann et al. (2008)
QacT	Proline and quaternary ammonium osmoprotectant	*Lactobacillus plantarum*	Wood et al. (2001)

Among the well-known amino acids with osmoprotectant properties, proline is one of the most commonly used by G− and G+ bacteria (Sleator and Hill 2002) (Tables 2 and 3). During osmotic stress, proline is either taken up from the environment or biosynthetized *de novo* in large quantities by bacterial cells (Kempf and Bremer 1998a). Interestingly, G+ bacteria primarily accumulate proline through biosynthesis (Table 2) (Tempest et al. 1970, Whatmore and Reed 1990), whereas G− bacteria typically achieve high proline concentrations through active transport from the environment (Table 3) (Brady and Csonka 1988, Sleator and Hill 2002).

Other amino acids commonly utilized by bacteria osmoprotectants are glutamine and glutamate. Under high osmotic stress, cytoplasmic glutamate levels notably increase in most bacteria groups, and in G− bacteria they can rise over 10-fold. Glutamine levels also increase, but its role is less significant due to its lower concentrations within cells and its function as a precursor for glutamate. In contrast, G+ bacteria typically have basal glutamate levels from 8 to 10 times higher than those in G−. Although G+ bacteria also exhibit an increase in glutamate content in response to osmotic stress, it is much subtler and slower than in G− bacteria (Csonka 1989, Saum et al. 2006).

#### Quaternary ammonium

Quaternary ammonium compounds (QACs) represent a diverse group of chemicals characterized by the presence of a nitrogen atom covalently bonded to four alkyl or aryl groups, making them permanently charged, cationic molecules across all pH values. One notable subset of this group includes methylated proline derivatives, where methyl groups are added to the proline’s pyrrolidine ring. This methylation can influence the biochemical properties of molecules, potentially affecting their hydrophobicity and solubility, and interactions with cellular components (Panday 2012, Yang et al. 2017). In particular, the methylation of amino acids affects protein function and interactions significantly (Bartuschat et al. 2019). Proline–betaine’s role in osmoregulation is well-documented, particularly in maintaining internal osmotic pressure and safeguarding cellular integrity under hypertonic stress (Bashir et al. 2014). Hydroxy proline–betaine, which features an added hydroxyl group (-OH) to proline–betaine, further increases its hydrophilicity and hydrogen bonding with water (Bashir et al. 2014).

#### Sugars

Sucrose and trehalose, nonreducing disaccharides, play pivotal roles in bacterial osmoregulation. Sucrose, composed of glucose and fructose linked by an α-(1→2) glycosidic bond, is highly soluble in water due to its nonreducing nature, involving both anomeric carbons in the bond (Ash 2017). This solubility is crucial for its function as an osmoprotectant, contributing to increase intracellular solute concentration under osmotic stress. Bacteria can either synthesize or uptake sucrose in response to osmotic stress, and its accumulation does not disrupt cellular biochemistry, as demonstrated by its protective effects against desiccation and osmotic stress in *E. coli* (Ash 2017). Trehalose, consisting of two glucose units linked by an α,α-(1→1) bond, is recognized for its stability and high water solubility, which are essential for its effectiveness in stabilizing proteins and membranes under stress conditions (Kosar et al. 2019). Its role extends beyond osmoprotection to maintain cell integrity against osmotic shock and dehydration, while preserving the native structure of biomolecules and preventing protein aggregation (Chen et al. 2017). In *Mycobacterium tuberculosis*, trehalose not only serves as an osmoprotectant, but also plays a role in cell wall construction and as a defense mechanism against host immune responses (Chen et al. 2017).

### Production of osmoprotectants by bacteria

The synthesis of osmoprotectants in microorganisms is often regulated by environmental stress signals, ensuring upregulation under osmotic stress conditions (Kempf and Bremer 1998a). As previously mentioned, the most commonly produced osmoprotectants by bacteria are glycine betaine, proline, trehalose, ectoine, and carnitine. G+/G− bacteria differ in their osmoprotective strategies, being G+ bacteria often more proficient at synthesizing these compounds *de novo* (Bremer 2000, Wood 2011).

Osmoprotectants are produced through amino acid or sugar biosynthetic pathways (Kanehisa and Goto 2000, Kanehisa 2019, Kanehisa et al. 2023). Microorganisms can use different sources of energy, carbon, and nutrient sources, including glucose, lactate, or yeast extract and various inorganic salts to produce osmoprotectants (Table 2). These compounds can be synthesized through genus- or even species-specific biosynthetic reactions, using precursors produced from intracellular metabolic processes like glycolysis and TCA cycle, or obtained from external sources (Jousse et al. 2017). For example, *B. subtilis* imports amino acids such as glutamic acid, l-glutamine, l-asparagine, l-aspartic acid, l-arginine, ornityne, and citruline, converting them to proline to increase osmotic stress (Stöveken et al. 2011). Similarly, glucose serves as a primary carbon source for the synthesis of trehalose and other sugars, such as mannose and sucrose (Elbein et al. 2003, Sudmalis et al. 2018).

Osmoprotectants can be extracted from bacteria by two main methods: cell lysis and osmotic shock. In the first case, cells are dissolved or lysed to obtain an extract, which then requires separation and purification to isolate the osmoprotectants of interest from the many other compounds present. The second case involves placing cells in a low-salt solution, causing osmoprotectants to be secreted through the cell’s transport channels, resulting in a relatively pure product. Qualitative and quantitative measurements of osmoprotectants are typically carried out using spectroscopic techniques, such as mass spectrometry coupled with various types of chromatography, nuclear magnetic resonance, or UV measurements. Additionally, dedicated enzyme assays can be employed for individual osmoprotectants. However, the lack of a gold standard for measurement and the consequent diversity of methodological approaches to measuring osmoprotectants has led to inconsistencies in the final units of osmoprotectant content in the samples tested (Table 2). This lack of standardization can create challenges when comparing osmoprotectants production across different strains.

### Transport of osmoprotectants

Another strategy that enables microorganisms to survive changes in osmotic pressure is the accumulation of osmoprotectants or their precursors from exogenous sources using specialized transporters that mediate their active uptake. Microorganisms have evolved a variety of transporters that allow importing nearly all classes of osmoprotectants (Tables 1 and 3). When bacteria can acquire osmoprotectants from their environment, they typically reduce or quit the endogenous synthesis of these compounds, as external acquisition is energetically more energy-efficient and supports better cellular homeostasis (Krämer 2010).

G+ and G− bacteria exhibit a wide variety of transport systems responsible for the import of osmoprotectants, differing in structure, mechanism of action, and substrate specificity (Brady and Csonka 1988, Sleator and Hill 2002, Hoffmann and Bremer 2011, 2017). These transporters belong to different families, such as ABC (ATP-binding cassette) transporters, ion gradient-dependent transporters and uniporters (Schulz et al. 2017, Teichmann et al. 2017). Each family is characterized by its unique molecular features and the specific way it uses energy to transport substances across the cell membrane (Table 3).

In G+ bacteria, such as *B. subtilis*, the betaine transporters OpuA, OpuB, OpuC, OpuD, and OpuE (Table 3), which are members of the ABC family, play a key role. These transport systems use energy from ATP hydrolysis to move osmoprotectants across the cell membrane, a process essential for adaptation to high-salt environments. In contrast, G− bacteria, such as *E. coli* and *Pseudomonas syringae*, use transport systems such as ProU, which is also a member of the ABC family, and BetT, which relies on the sodium gradient. These systems are representative, but there are many other specific uptake systems tailored to the needs of individual microorganisms. Table 3 provides a comprehensive overview of the most important osmoprotectants transport systems identified to date in G+ and G− bacteria. Notably, in the soil environment, the major bacterial families such as Proteobacteria, Firmicutes, and Actinobacteria often have similar osmoprotectant uptake capabilities. This shared ability underscores the importance of osmoprotectants transport for survival in diverse and osmotically challenging environments (Paul 2013).

Understanding the chemical nature of osmoprotectants in soil may enable a more accurate prediction of their impacts on soil properties. This knowledge provides a foundation for exploring how osmoprotectants influence soil ecosystems and the organisms within them, paving the way for the next discussion on their effects on soil properties and microbial life, which will be described in next chapters.

## Role of osmoprotectants in soil

The soil environment is a complex ecosystem with countless connections and interactions among microorganisms, macroorganisms, and plants. Soil components, including microorganisms, participate in the biogeochemical cycle of nutrients (e.g. carbon, nitrogen, phosphorus, and sulfur) and the water cycle. Soil acts as a specific carbon reservoir, storing organic matter and contributing to carbon sequestration. This process helps regulate atmospheric carbon dioxide levels in the atmosphere, which has an impact on the climate. Additionally, soil serves as a natural filter, purifying water passing through its layers, which is crucial for the protection of groundwater and surface water (Strawn 2021).

Salt accumulation leads to degradation of soil structure, reduced permeability, and water retention capacity (Tang et al. 2021). Consequently, degraded soil is more susceptible to water and wind erosion. As osmotic stress progresses, there can be a reduction in the number of stress-adapted plant species and microorganisms, potentially altering ecosystem structure and function. Agrobiotechnology and microbiology face the challenge of supporting farmers in these situations. Using knowledge of microorganisms’ ability to adapt to extreme environments, attempts can be made to assist plants and soils under stressful conditions. As previously described, one adaptive strategy found in microorganisms, algae, and plants is the production of osmoprotectants, a key protective mechanism found mainly in halophiles and halotolerant cells. The potential of these compounds has been documented in the literature, but the knowledge remains fragmented and their practical application in agriculture have yet to be fully explored.

The impact of osmoprotectants on soil properties is complex, reflecting the intricate interplay among soil physical, chemical, and biological characteristics. This interconnected nature is a key advantage of using osmoprotectants, as their addition can trigger multiple effects and mechanisms that can enhance soil conditions. Notably, the application of osmoprotectants results in a marked increase in both bacterial and fungal populations (Gouffi et al. 2000, George et al. 2015), among other effects, altering and elevating different metabolic pathways essential for the cycling of organic matters, nutrients, and minerals within the soil (Lucchesi et al. 1995).

**Figure 3.**
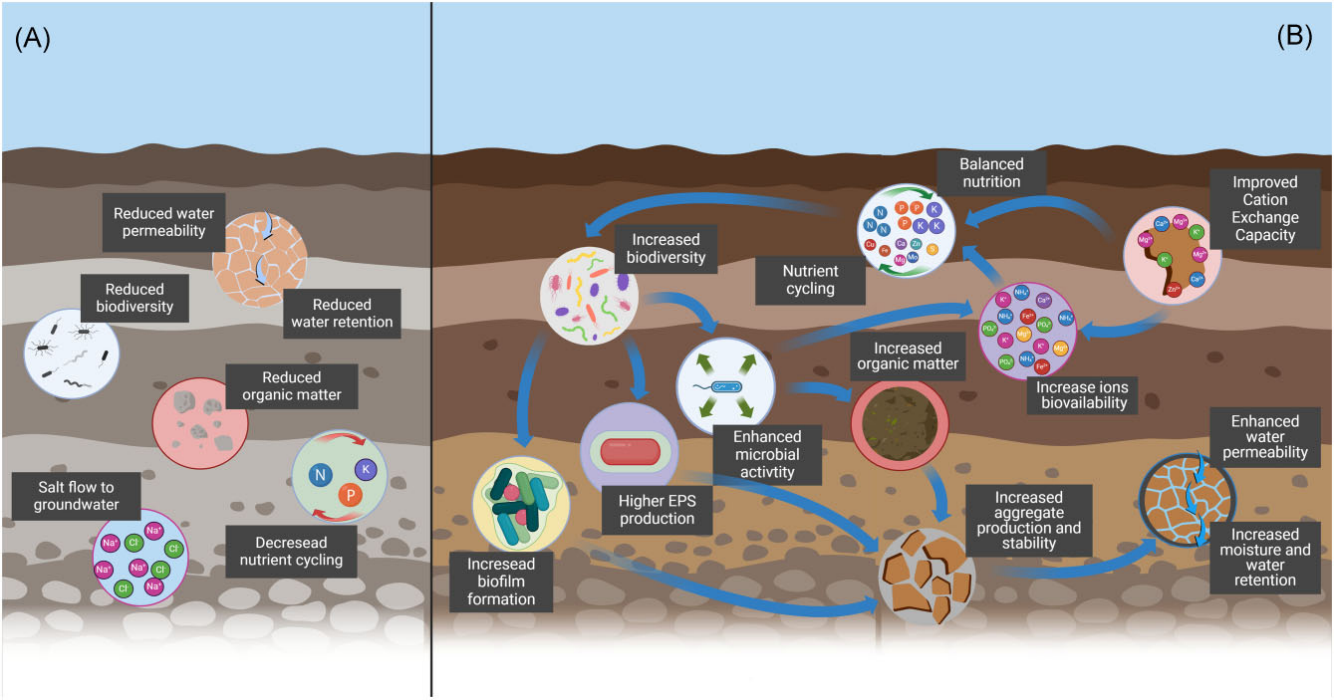
Effects of osmoprotectants supplementation on salinized soils. (A) Effect of salinity on soil. (B) Effect of supplementation of osmoprotectants or osmoprotectants-producing bacteria to the salinized soils.

Supplementation of saline soils with halotolerant bacteria can affect the availability of macro- and micronutrients (Fig. 3). For instance, *B. subtilis* BSN-1 produces high amounts of glutamic acid as an osmoprotectant, which can lower the pH of the soil matrix, thereby influencing the availability of compounds like phosphorus (Wang et al. 2021b). Liang et al. (2023), investigated the effects of adding a consortium of different halotolerant and plant growth promoting bacteria (*B. megaterium, Azospirillum brasilense, B. subtilis*, and *Paenibacillus mucilaginosus*), known for their osmoprotectant producing capabilities, to salinized soils (Gal and Choi 1998, Kempf and Bremer 1998b). This approach provides a holistic approach, by utilizing the diverse functions of these bacteria. The study reported increases in soil moisture content, total phosphorus, total nitrogen, and soil organic matter (Liang et al. 2023) (Fig. 3). These chemical and physical changes likely contributed to shifts in autochthonic bacterial consortium composition. At the phylum level, the relative abundance of *Proteobacteria* increased by 14.28%, *Bacteroides* content grew, and *Firmicutes* and *Actinobacteriota* decreased by 4.38% and 5.19%, respectively. At the class level, increases were noticed in the relative abundance of Alphaproteobacteria, Gammaproteobacteria (Proteobacteria), and Bacteroidia. At the family and genus levels, there was an increase in the families within Proteobacteria, e.g. *Rhodobacteraceae* and *Rhizobiaceae*, as well as in *Bacteroidota* families, such as *Marinilabiliaceae*, also increased (Liang et al. 2023).

The beneficial effects of various osmoprotectants and their impact on different organisms are still under investigation. Gouffi et al. (2000) studied *S. meliloti*, showing that pipecolic acid can provide osmoprotective effects. Mishra and Sharma (2012) found that osmoprotectants such as glycine, proline, betaine, and glycerol significantly enhance the salinity tolerance of rhizobacteria, with yeast extract showing the highest osmoprotective effect. Gal and Choi (1998) reported that osmoprotectants like proline and GB improve the growth and nitrogenase activity of *Rhizobium* and *Azospirillum* under osmotic stress, with GB being the most effective. Iskandaryan (2023) investigated the role of GB in stimulating growth and hydrogenase activity in the bacterium *Ralstonia eutropha* H16, highlighting its potential to enhance microbial metabolic activities in soil (Iskandaryan 2023).

According to Beshay and Daba (2009), various substances commonly recognized as osmoprotectants, including xylose, galactose, glycerol, glucose, fructose, sucrose, mannose, and starch, have been shown to increase EPS production (Beshay and Daba 2009). Research involving *Pseudomonas aeruginosa* has tested the effect of the use of osmoprotectants on the remediation of salinized areas contaminated with hydrocarbons and aromatic compounds. Bazaire et al. (2007) used GB, which resulted in higher biofilm formation and increased rate of benzoate degradation (Bazire et al. 2007) (Fig. 3). Elevated EPS content in soil is associated with enhanced aggregate production and stability (Beshay and Daba 2009, Pandey et al. 2020). Enhanced aggregate stability is crucial for reducing soil erosion and improving water infiltration, thereby increasing the soil’s water-holding capacity, particularly in sandy or coarse-textured soils (Vermang et al. 2009). These changes are attributed to the hygroscopic nature of EPS, which helps retain moisture within the soil matrix, thus enhancing the binding of soil particles (Mugnai et al. 2018). This property is vital during periods of low precipitation, as it aids in sustaining soil moisture levels and plant growth. EPS are a crucial element of biofilm, a critical aspect of soil and plant microbiology. Trehalose, for example, has been associated with improved biofilm formation by bacteria. The effect of biofilms on soil properties is multifaceted, influencing various aspects such as soil strength, water retention, and nutrient dynamics. Biofilms enhance water permeability, and fortify internal cohesion of soil macroaggregates (Fig. 3), all of which benefit plant growth and soil structure (Anju et al. 2021). They also increase the shear strength of granular soils, with treated samples exhibiting greater peak stress than untreated ones due to biofilm-induced densification (Al-Awad 2018). Shariq et al. (2021) demonstrated that biofilms enhance sand strength across various moisture levels, suggesting that higher concentrations of biofilm can be an effective method for soil stabilization under different moisture conditions (Shariq et al. 2021). Additionally, phototrophic biofilms have been shown to transform dissolved organic matter (DOM) in the soil, increasing the abundance of labile DOM and enhancing soil fertility (Liu et al. 2023a).

The chemical properties of soil are fundamental to its fertility and the overall health of the supported ecosystem. The application of osmoprotectants can influence these properties in several ways, contributing to nutrient availability, altering soil pH, and affecting the cation exchange capacity (CEC), all of which are essential for plant growth and microbial activity. Osmoprotectants in soil can indirectly impact CEC, a measure of the soil’s ability to retain and exchange cationic nutrients. Research has shown that halophilic bacterial biofilm combined with rice (*Oryza sativa* L.) husk can effectively reduce sodium levels in saline water, highlighting biofilms’ potential to alter soil salinity and improve cation exchange processes (Ahsan et al. 2023). Finally, Çam et al. (2022) found that salinity-resistant *Azotobacter* spp. biofilms significantly improve maize (*Zea Mays* L.) growth under salt stress, underscoring the role of biofilms in modifying soil ion exchange and enhancing plant resilience (Çam et al. 2022).

Although the application of exogenous osmoprotectants and halotolerant bacterial strains has demonstrated numerous benefits for soil properties and plant resilience, it is important to acknowledge potential risks associated with their introduction into natural soils. One significant concern is the possible disruption of indigenous microbial communities. The addition of nonnative strains or compounds can lead to shifts in microbial diversity, competition for ecological niches, and even suppression of beneficial native taxa (Manfredini et al. 2021). These effects may result in unintended alterations to essential ecosystem services, such as nutrient cycling or pathogen suppression (Trabelsi and Mhamdi 2013). Furthermore, the long-term persistence and ecological behavior of introduced strains remain largely unpredictable in many cases. Therefore, prior to large-scale application, it is crucial to assess the compatibility of microbial inoculants with local soil microbiota through microbiome profiling and functional assays (Manfredini et al. 2021).

## Role of osmoprotectants in plants

Like bacteria, plants exposed to abiotic stresses such as salinity and drought, rely on osmoprotectants to maintain cell turgor and facilitate water uptake gradients (Kido et al. 2019). As climate change continues to exacerbate these stresses, there is an urgent need to develop new adaptive strategies in agriculture to support plant productivity. Although plants naturally produce osmoprotectants, their production is often insufficient to counteract severe stress conditions (Khan et al. 2015b). Genetic engineering has enabled the introduction of osmoprotectants pathways into plants, significantly enhancing their stress tolerance. However, these transgenic plants exhibit varying levels of tolerance due to multiple metabolic limitations (Rontein et al. 2002, Zulfiqar et al. 2020).

Consequently, researchers have explored the use of exogenous osmoprotectants, applied either directly to the soil or as foliar sprays on plant leaves during periods of stress (Ashraf and Foolad 2007). For instance, the addition of proline and GB to the soil increases proline and nitrogen content in soybean leaves, leading to improved seed yield under drought conditions (Ayman et al. 2016). Similarly, applying bacterial osmoprotectants can enhance plant resistance to drought (Jha et al. 2011). Therefore, employing bacteria capable of producing osmoprotectants as bioinoculants emerges as a promising strategy to bolster the resilience of plants vulnerable to abiotic stresses (Fouda et al. 2021).

### Naturally occurring osmoprotectants in plants

Plants naturally synthesize osmoprotectants like amino acids (proline and ectoine), QACs (GB, choline-O-sulfate), and sugar alcohols/nonreducing sugars (trehalose, sorbitol, and mannitol). These compounds are primarily localized within cellular compartments such as the cytosol, chloroplasts, and mitochondria, but the precise compartmentalization of some of them still remains to be fully elucidated. The latest research conducted in the 1980s, now requires reassessment using contemporary methods for organelle separation and precise quantification of osmoprotectants.

To enhance stress resilience in agricultural crops, it is essential to know the precise cellular localization and the specific functions of individual osmoprotectants. Amino acids play a central role in helping plants survive under abiotic stress conditions such as drought and salinity (Joshi et al. 2010). When plants face these stresses, they accumulate various proteinogenic amino acids, including proline, alanine, arginine, glycine, and branched-chain amino acids like isoleucine, leucine, and valine (Mansour 2000, Araújo et al. 2010, Woodrow et al. 2017, Carillo 2018). Among them, proline is especially notable for its role in plant defense and metabolism, acting as a critical osmoprotectant, as similarly reported in bacteria.

#### Amino acids: spotlight on proline

Proline synthesis in plant cells occurs through two distinct pathways: the glutamate pathway and the ornithine pathway (Rai and Penna 2013). The glutamate pathway mediated by the sequential action of Δ¹-pyrroline-5-carboxylate synthetase [P5CS] and pyrroline-5-carboxylate reductase 1 [P5CR] is considered the primary route for proline accumulation and takes place in the cytosol and chloroplasts. P5CS gene encodes for a bifunctional enzyme responsible for catalyzing two steps in proline biosynthesis, while P5CR catalyzes the final step, converting glutamate and ornithine into proline. In *Arabidopsis*, both P5CS and P5CR genes are crucial for viability. Double mutations in P5CS1 and P5CS2 are gametophytic lethal, resulting in the failure to form fertile p5cs1/p5cs2 mutant pollen, while homozygous P5CR mutants produce embryos that fail to develop beyond the very early stages, and are ultimately aborted (Funck et al. 2010, Mattioli et al. 2012). These findings indicate that no alternative pathway can produce adequate proline levels required for successful sexual reproduction. In contrast to the glutamate pathway, the ornithine pathway is activated under nitrogen-limiting or osmotic stress conditions in chloroplasts (Delauney et al. 1993, Winter et al. 2015, Dar et al. 2016). This process helps balance the low NADPH:NADP+ ratio, sustains electron flow between photosynthetic excitation centers, regulates redox balance, alleviates cytoplasmic acidosis, and protects against photoinhibition and damage to the photosynthetic apparatus (Filippou et al. 2014), while the catabolism of proline in mitochondria contributes to oxidative respiration and produces energy for resumed plant growth (Kaur and Asthir 2015). Proline acts as a metabolic signal, stabilizing metabolite pools and thereby exerting a beneficial impact on growth and development (Verbruggen and Hermans 2008).

The concentration of free proline within a plant cell is primarily influenced by four metabolic pathways: biosynthesis, degradation, protein biosynthesis-mediated consumption, and protein degradation-induced release (Hildebrandt 2018). It also varies across different plant organs, typically being higher in reproductive organs than in vegetative tissues. This variation extends to different organelles and subcellular structures, where proline distribution is heterogeneous. High levels of proline have often been observed in plant organs during internally regulated dehydration processes, such as in seeds or pollen. Moreover, a notable leaf-to-root proline ratio has been documented in various species, including *Arabidopsis*, lentil, and common bean (Misra and Saxena 2009). Furthermore, it has been reported that in *Vicia faba* and *Brassica juncea* the proline content in leaves decreases with maturation, while, proline distribution within the leaf blades of *Chrysanthemum indicum*, is uneven, a pattern also found between the lower epidermis and other leaf parts in *Arabidopsis*.

Proline is delivered throughout the plant via long-distance transport through vascular tissues and local transport mediated by plasma membrane permeability or intercellular connections via plasmodesmata. Although the nature of organellar proline transporters remains unclear, several plasma membrane-localized proline transporters have been identified. Notably, various members of the amino acid/auxin permease family facilitate amino acid-proton symport (Dinkeloo et al. 2018, Yang et al. 2020). Within this group, the amino acid permease (AAP) and lysine histidine transporter (LHT) subfamilies transport proline along with a wide array of both neutral and charged amino acids (Fischer et al. 1995, Hirner et al. 2006). Conversely, the proline transporter (ProT) subfamily displays more selective substrate specificity, transporting proline, glycine betaine, and γ-aminobutyric acid (GABA) (Lehmann et al. 2011). Studies on *Arabidopsis* aap1 mutants indicate that AAP1 may play a role in proline uptake from the growth medium (Perchlik et al. 2014, Wang et al. 2017), while the Siliques Are Red1 (SIAR1/UMAMIT18) protein has been identified in *Arabidopsis* as the first known transporter capable of bidirectional amino acid transport, with its directionality dependent on the electrochemical gradient across the membrane (Ladwig et al. 2012). SIAR1 belongs to a large protein family consisting of 44 members in *Arabidopsis*, several of which have been characterized as broad-specificity amino acid exporters and referred to as “usually multiple amino acids move in and out transporters” (UMAMITs) (Müller et al. 2015, Besnard et al. 2016).

Several studies have reported the accumulation of proline, particularly in the cytoplasm and chloroplast (Verbruggen and Hermans 2008), in response to various stresses like drought (Kumar et al. 2022), salinity (Akram et al. 2012, Vives-Peris et al. 2017), metal toxicity (Zouari et al. 2016), and high temperature (Akram et al. 2012). This accumulation can result from either increased synthesis or reduced degradation of proline (Verbruggen and Hermans 2008), which helps protect cells against ROS (Handa et al. 2018). The extent of proline accumulation can vary significantly across species, with concentrations potentially increasing up to 100-fold under stress compared to normal conditions (Verbruggen and Hermans 2008).

#### QACs: focus on glycine betaine

With respect to QACs, such as GB and choline-O-sulfate, these are synthesized from choline through a two-step oxidation process involving choline monooxygenase and betaine aldehyde dehydrogenase. These compounds are vital for maintaining the osmotic balance within cells and protecting cellular components against denaturation. GB, in particular, is a widely distributed osmoprotectant. Being an electrically neutral dipolar molecule at physiological pH, it increasingly accumulates in plants under abiotic stress conditions (drought, high temperature, and salinity) and contributes to increased cell osmolality. The extent of GB accumulation varies depending on the plant species and its degree of stress tolerance, with the highest levels typically found in leaves, although influenced by the leaf age (Yamada et al. 2009). GB synthesis primarily occurs in the cytoplasm, and, in dicots, also in chloroplasts, while in monocots, it takes place in peroxisomes (Nakamura et al. 1997, Mitsuya et al. 2011). This process mainly involves the conversion of choline through oxidation steps catalyzed by choline monooxygenase (EC 1.14.15.7) and betaine aldehyde dehydrogenase (BADH EC 1.2.1.8) (Rathinasabapathi et al. 1997). The localization of BADH isoenzymes varies among plant species. For instance, in spinach, distinctive isoenzymes target chloroplasts and cytosol; in barley (*Hordeum vulgare* L.), they are located in peroxisomes and cytosol; in rice (*O. sativa* L.), they are confined to peroxisomes. Additionally, *Avicennia marina* exhibits a unique distribution with one isoenzyme present in chloroplasts and another in peroxisomes (Weigel et al. 1986). Halophilic plants and microorganisms, as well as methanogenic organisms, can produce GB from glycine, as process catalyzed by two crucial enzymes: glycine sarcosine methyl transferase and sarcosine dimethylglycine transferase (Waditee et al. 2005). In angiosperms, GB synthesized in chloroplasts protects membranes, enzymes, and key proteins involved in photosynthesis, such as Rubisco and PSII, under harsh environmental conditions. Additionally, when GB is supplied exogenously to older parts, it is rapidly retranslocated to younger, expanding tissues, where it plays a critical role in safeguarding these areas. However, some species such as rice, tomato (*Solanum lycopersicum L*.) and tobacco (*Nicotiana tabacum* L.), and major cereals like maize, wheat (*Triticum aestivum* L.), and barley (*H. vulgare* L.) have a limited capacity to naturally accumulate GB (Fariduddin et al. 2013, Kurepin et al. 2015). In this respect, genetic engineering to identify and transfer genes involved in GB biosynthesis has emerged as a promising strategy for enhancing stress tolerance in nonbetaine accumulator crops (Chen and Murata 2011). This approach has proven effective across various crop species, as evidenced by the successful development of transgenic cultivars.

#### Sugar alcohols and nonreducing sugars

Sugar alcohols and nonreducing sugars, like mannitol, sorbitol, and trehalose, produced from simple sugar phosphates, are essential for the proper cellular osmotic adjustment because they create amorphous protective structures that prevent dehydration and stress-related damage to cell components. Additionally, they scavenge ROS, stabilize proteins and membranes, and shield the plant from oxidative stress.

Mannitol is a pivotal osmoprotectant that accumulates in various plant species (Slama et al. 2015), playing a crucial role in photosynthesis and abiotic stress tolerance (Loescher et al. 1992). As an osmoprotectant, mannitol helps counteract ROS and serves as a repository of reducing power. However, not all plant species naturally accumulate mannitol. Thus, integrating mannitol biosynthesis genes from other organisms into crop plants offers a promising strategy to enhance their stress tolerance and mitigate the adverse impacts of climate change.

Trehalose is a disaccharide composed of two glucose units linked by an α,α-1,1-glycosidic bond. This compound is synthesized across a wide range of organisms, including bacteria, fungi, nematodes, arthropods, and plants, yet it is absent from vertebrates. The plant trehalose was first discovered in *Selaginella lepidophylla* by Anselmino and Gilg (1913). Although most higher plants contain only trace amounts of trehalose, notable exceptions include the resurrection plant *S. lepidophylla*, which is highly stress-tolerant. As a cell protectant, trehalose preserves protein structure and lipid membrane integrity during exposure to environmental stresses like dehydration, extreme temperatures, cold, heat, and oxidative stress (Elbein et al. 2003). It maintains stability under conditions of high temperature up to 100°C and across a wide pH spectrum for extended periods. Anhydrobiotic organisms including yeast, fungi, resurrection plants, nematodes, rotifers, and brine shrimp cysts are rich in trehalose, which is integral to their ability to endure prolonged desiccation without water (Richards et al. 2002). The capacity for survival in these organisms under anhydrobiotic conditions indeed correlates with the production of trehalose.

### Transgenic approaches to increase plant tolerance to osmotic stress

Traditional plant-breeding techniques and chemical interventions may no longer be sufficient to enhance crop tolerance or resilience to address global climate challenges, necessitating more innovative approaches to meet pressing agricultural needs. Consequently, the transgenic approach has been developed to produce crop varieties more tolerant and/or resilient to biotic and abiotic stresses (Khan et al. 2015a). Control of certain biotic stressors has been achieved through the manipulation of single genes, while managing most abiotic stresses typically requires the involvement of multiple genes. A common strategy in developing transgenic lines involves upregulating genes associated with the synthesis of osmoprotectants (Paul and Roychoudhury 2018). Several plant species have been genetically engineered with multiple genes to enhance their stress tolerance. In *Arabidopsis*, genetic engineering has increased resilience to osmotic stress through the upregulation of proline-related genes. Similarly, transgenic tobacco (*N. tabacum L*.) expressing the A1-Pyrroline-5-Carboxylate Synthetase (P5CS) gene from *Vigna aconifolia* (Jacq) increased proline synthesis and root biomass under water stress, while transgenic potatoes (*Solanum tuberosum L*.) expressing the P5CS gene from *Arabidopsis* spp. (Kishor et al. 1995, Hmida-Sayari et al. 2005) and *Medicago sativa* L. overexpressing the dehydration-responsive element binding protein GmDREB6 (Yamada et al. 2005) accumulated significantly higher proline levels under salt stress compared to control plants. Transgenic petunia (*Petunia hybrida* cv. “Mitchell”) plants with P5CS genes from *Arabidopsis thaliana* or *O. sativa* also exhibited increased proline levels that resulted in sustained drought resistance over a 14-day period compared to wild-type plants under normal conditions (Yamada et al. 2005). Further research by Zhang et al. (2014) and Surekha et al. (2014) confirmed that transgenic tobacco and pigeon pea (*Cajanus cajan* L.) plants expressing proline synthesis genes experienced less oxidative damage and maintained higher proline contents under abiotic stress conditions.

In addition to proline, transgenic approaches have focused on the trehalose biosynthetic pathway using genes sourced from prokaryotes and different crops (Zentella et al. 1999, Lunn et al. 2014), with transgenic plants showing significant increases in drought tolerance (Yeo et al. 2000). Furthermore, *Arabidopsis*, rice, eucalyptus (*Eucalyptus obliqua*), wheat, tobacco, tomato, and potato transgenics overexpressing GB biosynthetic genes have shown increased GB accumulation and enhanced stress tolerance (Huang et al. 2000, Kurepin et al. 2017, Tian et al. 2017, Zhang et al. 2019). These plants not only survived stressful conditions but also exhibited improved reproductive features, including increased flower and fruit production (Sulpice et al. 2003, Park et al. 2004). Furthermore, transgenic sweet potato lines engineered to express the BADH gene showed increased stress tolerance through enhanced cell membrane integrity and photosynthetic capacity, along with reduced ROS accumulation (Fan et al. 2012).

### Microbial allies: plant stress alleviation

**Figure 4.**
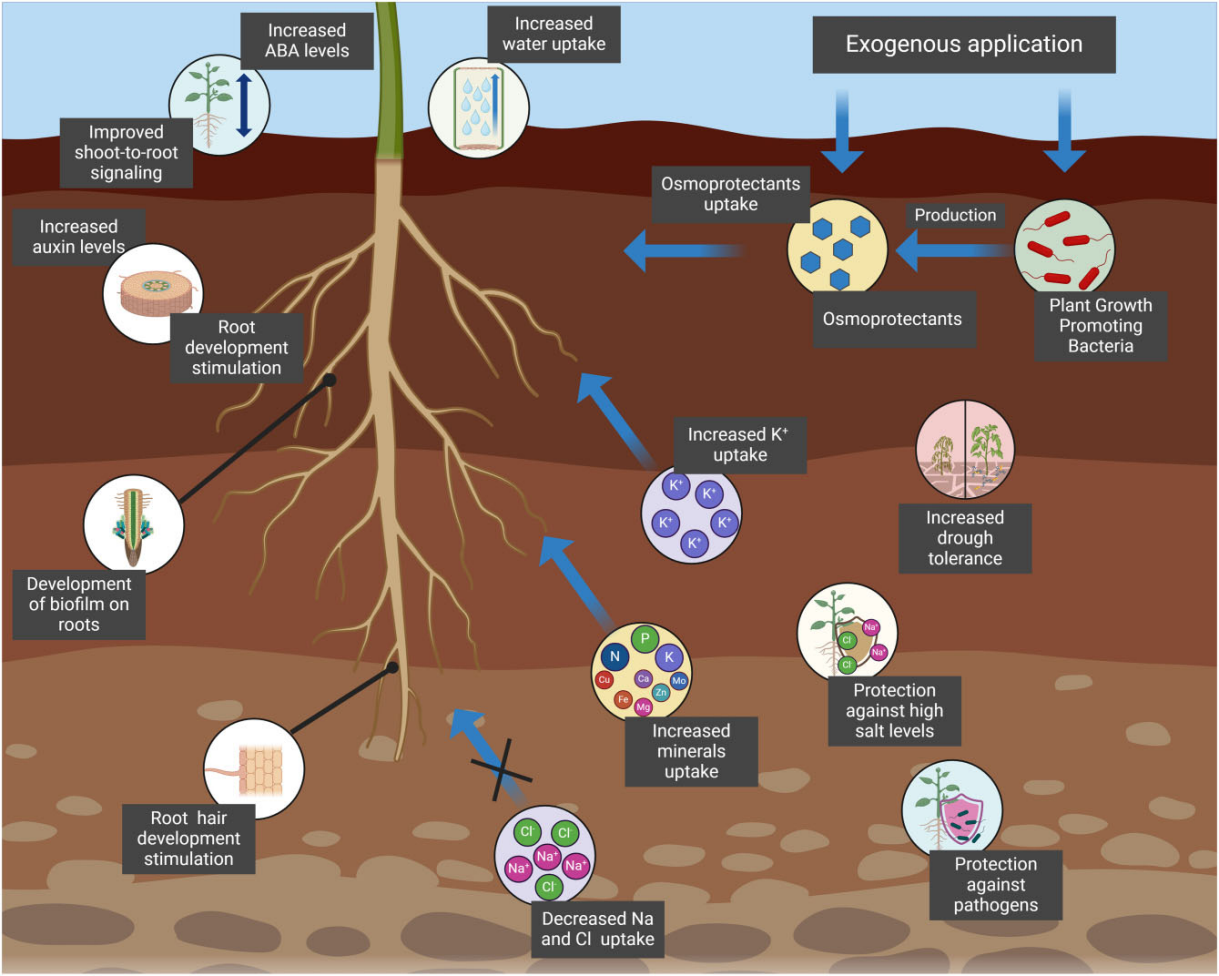
Effects of osmoprotectants and halotolerant bacteria bioaugmentation at the soil–plant interface.

Plants maintain beneficial relationships with bacteria that aid their survival under abiotic stress conditions. Plant growth-promoting bacteria (PGPB) are essential components of natural and agricultural ecosystems, inhabiting various ecological niches such as the root system, rhizosphere, phyllosphere, and spermosphere. These microbes establish complex, symbiotic interactions with plants, affecting their physiological and molecular mechanisms, which include: osmolyte synthesis, modification in the root system, induction of the antioxidant machinery, production of extracellular exopolymers and siderophores, modulation of phytohormones, uptake of minerals, and control of phytopathogens. The application of PGPB, particularly their subgroup plant growth-promoting rhizobacteria (PGPR) that colonize the rhizosphere, has garnered significant interest among researchers as a strategy to balance crop productivity with soil health and fertility (Kumar et al. 2020) (Fig. 4). This approach involves isolating PGPB from various ecosystems with environmental constraints such as salinity, alkalinity, acidity, and aridity.

PGPR assist plants in accumulating low molecular weight osmolytes, such as soluble sugars, amino acids, quaternary amines, polyols, and tetrahydropyrimidines (Fig. 4) under osmotic stress. Research indicates that, rather than synthesizing these osmolytes *de novo*, plants prefer to absorb them from high-tolerant PGPR, especially under high salt conditions (Zhu et al. 2015). Poststress, osmolytes aid in repairing plant tissues and serve as sources of nitrogen and energy (Kumar Arora et al. 2020).

**Figure 5.**
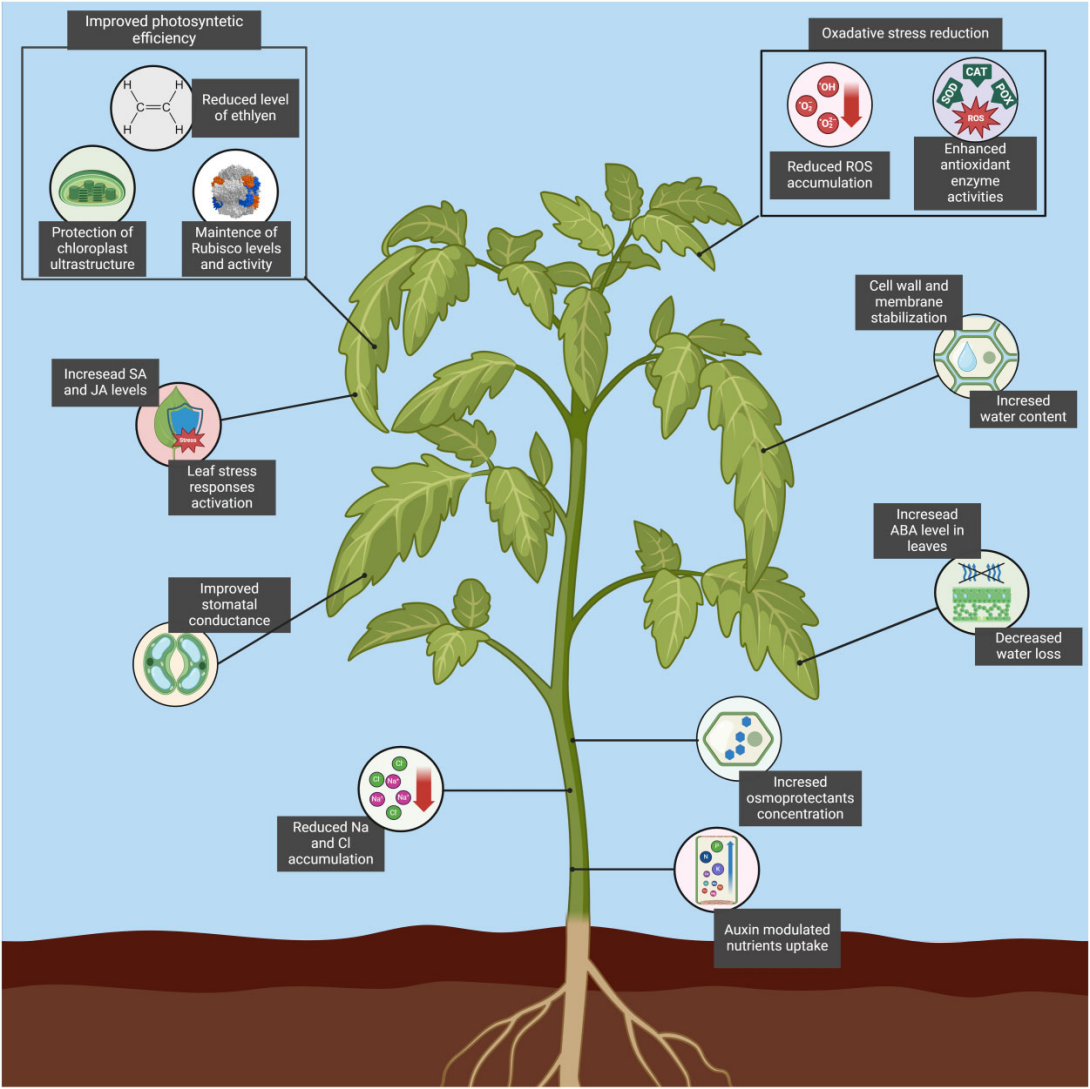
Effects of the addition of exogenous osmoprotectants on the plant physiology, with an emphasis on its response to osmotic and salt stress.

Drought-tolerant PGPR have been shown to enhance biomass and water potential in stressed maize plants, reduce water loss, decrease antioxidant activity, and increase the levels of osmoprotectants like proline, free amino acids, and sugars (Fig. 5) (Vardharajula et al. 2011). Inoculation of soybean plants (*Glycine max* L.) with *Pseudomonas putida* under water stress results in enhanced chlorophyll content, shoot length, and biomass (Kang et al. 2014) (Fig. 5). Similarly, rice plants inoculated with *P. pseudoalcaligenes* and *Bacillus pumilus* show improved salinity tolerance and higher concentrations of GB (Bano and Fatima 2009, Jha et al. 2011). PGPR like *Rhizobium* and *Pseudomonas*, alleviate salt stress in NaCl-affected maize plants by reducing electrolyte leakage, osmotic potential, while enhancing proline production and selective uptake of potassium ions (Fig. 5). Among these, *Rhizobium* species are particularly well-known for their role as symbiotic nitrogen-fixing bacteria that form mutualistic associations with legumes, contributing significantly to sustainable agriculture through biological nitrogen fixation. Under saline conditions, their survival and symbiotic efficiency are supported by the production of compatible solutes such as trehalose, ectoine, and proline, which help maintain cellular osmotic homeostasis (Basu et al. 2023, Muntyan and Roumiantseva 2024). For instance, *S. meliloti* can upregulate genes involved in trehalose biosynthesis in response to salt stress, enabling the bacterium to survive and function in saline environments (Muntyan and Roumiantseva 2024). Importantly, rhizobia can retain nitrogen-fixing activity even under osmotic stress, as evidenced by the sustained expression of the *nifH* gene, which encodes a key component of the nitrogenase complex responsible for atmospheric nitrogen reduction (Win et al. 2023, Gaied et al. 2024). This capacity not only supports plant nitrogen nutrition under adverse conditions but also reduces the need for synthetic nitrogen fertilizers in saline soils. El-Akhal et al. (2013) demonstrated that inoculation with salt-tolerant rhizobial strains enhanced legume growth and vigor under saline conditions to levels comparable with nitrogen-fertilized plants.

Enhanced accumulation of osmoprotectants in plants is also facilitated by rhizosphere bacteria producing EPS (Fig. 4). As previously mentioned, EPS are vital for biofilm formation (Naseem et al. 2018). Bacteria such as *Pseudomonas, Azospirillum*, and *Rhizobium* produce EPS, which have been shown to enhance plant resistance to both salinity and drought. For instance, *P. putida* forms biofilms on sunflower (*Helianthus annuus* L.) roots under water-deficit conditions, while *Bradyrhizobium* improves drought tolerance in cowpeas (*Vigna unguiculata* L.) (Khan and Bano 2016). Additionally, *Pseudomonas stutzeri* enhances salt tolerance in chilli peppers (*Capsicum annuum* L.) (Bacilio et al. 2016), while biofilm-forming microbes mitigate salinity stress in barley (Kasim et al. 2016).

A promising and highly effective approach to enhance plant resistance to abiotic stress involves the inoculation of plants with both endophytic and rhizospheric bacteria (Jha et al. 2011). A comparative study of these PGPR has found endophytes particularly more effective. Specifically, research on tomatoes revealed that endophytic bacteria, due to their intimate interaction with host tissues, outperform rhizospheric bacteria in several key areas: root hair development, osmoprotectants synthesis, siderophores, and hormone production. This close association with plant tissues leads to more evolved mutualistic interactions, enhancing plant resilience. Additionally, endophytes often act as biocontrol agents by competing with phytopathogens, further supporting plant health (Abbamondi et al. 2016).

Plants inoculated with PGPB demonstrate a high capacity for osmolyte accumulation and exhibit stimulatory effects on growth. Consequently, these bacterial strains offer a promising and cost-effective alternative for improving crop productivity and advancing sustainable agricultural practices. This is particularly important considering that, although the exogenous application of osmoprotectants such as proline or GB may seem like a straightforward approach to boosting plant growth and resilience against abiotic and biotic stresses, relying solely on these external osmolytes may not be economically viable in the long term perspectives.

## Osmoprotectants in agrobiotechnology—present

Current research in agricultural science, particularly concerning salinized soils, primarily focuses on developing self-sustaining plant cultivars capable of thriving under adverse conditions (Conti et al. 2022). Through selective breeding or engineering salinity tolerance, plants can be developed to produce higher levels of osmoprotectants, enhancing their survival and productivity even in challenging environmental settings (Rao et al. 2020, Wani et al. 2020). However, due to the regulatory constraints on genetically modified crops in many countries, the development of salt-resistant crop varieties often relies on techniques like directed evolution, which are drastically more time-consuming than for example biotechnological approaches, such as plant methylation (Rao et al. 2020). On the other hand, the generation of new cultivars using more traditional breeding methods is a lengthy process, often taking years to complete the necessary testing and meet regulatory standards.

Metabolic engineering of osmoprotectants has shown promising results in improving salt stress tolerance in crops (Omari Alzahrani 2021). As mentioned in a previous section, the most common approach is the modification of genes responsible for proline production, given its crucial role in stress adaptation. These genetic modifications vary across studies, targeting resistance to low temperatures, drought, or salt stress (Khan et al. 2015b). The most frequently modified transgenes are from the P5C gene family, from species like *A. thaliana, Daucus carota, N. tabacum*, and *S. tuberosum* (Kishor et al. 1995, Zhu et al. 1998, Sawahel and Hassan 2002, Han and Hwang 2003, Zhang et al. 2014, Khan et al. 2015b).

Another strategy involving osmoprotectants is seed priming. Drought in field conditions can significantly restrict water absorption and seed imbibition, thus there is a pressing need to develop methods that address these issues. Research on sorghum has demonstrated that seed priming with osmoprotectants can enhance both germination and early seedling growth under drought (Ojewumi 2023). Seed priming can also modulate enzyme activities essential for seed metabolism, while enhancing cellular defense mechanisms by reducing imbibition time, increasing the level of germination promoters, and regulating osmotic stress (Marthandan et al. 2020). Different types of priming techniques such as water-based, plant growth regulator-based, osmotic solution-based, chemical-based, and so on, are widely used to enhance drought tolerance in many crops (Marthandan et al. 2020). However, osmo-priming can occasionally lead to reduced seeds longevity. For instance, in tomato seeds, osmo-priming downregulates stress response genes, significantly shortening seeds storage time by >5-fold, causing osmo-primed seeds fail to germinate after 25 days, whereas unprimed seeds remain viable for up to 150 days (Petronilio et al. 2021).

An emerging approach gaining attention involves the use of biostimulants containing osmoprotectants, which are supplemented to salinized soils to alleviate abiotic stress in plants (Jiménez-Arias et al. 2021). A study on soybeans demonstrated that a biostimulant containing GB improved drought tolerance by enhancing physiological and biochemical responses, such as increased activities of antioxidant enzymes like superoxide dismutase, catalase, and ascorbate peroxidase. The treatment also increased proline accumulation, leaf photosynthetic rate, stomatal conductance, and transpiration rates, which are critical processes for osmotic adjustment (Repke et al. 2022). Hafez et al. (2021) studied the effects of combining biochar-based soil amendment with exogenous GB on rice under salt-affected soil conditions with varying irrigation intervals over two growing seasons. This treatment significantly reduced soil pH, electrical conductivity, and ESP, while increasing K^+^ accumulation in the leaves. Additionally, it improved the photosynthetic pigments’ content, and physiological attributes (net photosynthetic rate, stomatal conductance, and relative water content), and reduced electrolyte leakage in osmotic-stressed plants. Changes in the activity of antioxidant-related enzymes (catalase, ascorbate peroxidase, and peroxidase) were also observed, contributing to increased yield components, biological yield, harvest index, and nutrient value of rice grains (Hafez et al. 2021).

Currently, foliar application is the most widely used method for supplementing osmoprotectants to plants, enabling direct uptake through the leaves, thus leading to rapid physiological and biochemical responses that improve stress tolerance by maintaining cellular homeostasis, boosting photosynthetic efficiency, and reducing oxidative damage. Osmoprotectants can easily penetrate the leaf cells due to their high solubility and small molecular size. For example, GB is readily absorbed by plant cells when applied foliarly, diffusing through the cuticle and entering the cells via passive transport mechanisms. Foliar application of GB improves salt and drought tolerance in tomatoes by restoring the photosynthetic rate of salt-stressed plants to control levels, increasing stomatal conductance for efficient gas exchange, protecting chloroplast ultrastructure, and preventing the decrease of chlorophyll content and Rubisco activity (Mäkelä et al. 2000, Mäkelä 2004). GB foliar application also enhances relative water content under drought stress, particularly during the vegetative growth stage (Rezaei et al. 2012), and can increase chilling tolerance in tomato plants (Park et al. 2006). A foliar spray of 400 mg/l (3.41 mM/l) of GB onto a salt-sensitive cultivar of maize also enhances proline accumulation under salt stress conditions, maintaining transpiration efficiency and high photosynthetic performance (El-Newelny 2022).

Similar to GB, proline is efficiently absorbed through the concentration gradient across the leaf surface and, once inside the plant, accumulates where it is most needed to mitigate stress effects. This uptake is facilitated by plant transport proteins located in the leaf cell plasma membranes, which belong to four transporter families found in *A. thaliana* and, such as AtProT family transporters (AtProT1, -2, and -3), AAPs (AtAAP2, AtAAP5, and AtAAP6), lysine/histidine transporters (AtLHT1), and cationic amino acid transporters (CATs) with the representative protein AtCAT1 aiding in the movement of osmoprotectants into the cytoplasm, with some being rapidly translocated to meristems and roots (Yang et al. 2020).

Another method involves the postharvest application of osmoprotectants to plants, fruits, and cuttings. As an example, treating cut roses with l-proline can enhance water fluxes, water conductivity, relative water content, and stomatal conductance in comparison to untreated plants (Di Stasio et al. 2018). This treatment helps cut plants remain fresh longer before wilting. In the context of fruit treatment with osmoprotectants, spraying GB on sweet cherries (*Prunus avium* L.) enhanced fruit quality and reduced storage disorders, with preharvest application yielding more pronounced benefits. Postharvest GB application did not significantly affect most quality parameters of fruits, but did result in fruit softening and reduced decay. Preharvest GB application, on the other hand, was more effective, improving fruit firmness and reducing peduncle browning and pitting, and when applied three times, further enhanced firmness, titratable acidity, and reduced pitting. Although it slightly reduced fruit size, it also increased calcium uptake and resulted in firmer flesh (Li et al. 2019).

In conclusion, the application of osmoprotectants in agricultural science offers a multifaceted approach to enhancing crop resilience in salinized soils. However, continued interdisciplinary research and technological advancements are essential to fully harness the benefits of osmoprotectants and establish novel and sustainable applications of these compounds.

## Prospects for osmoprotectants in agrobiotechnology

Although osmoprotectants show promise in enhancing plant resilience, promoting beneficial microbial activity, and improving soil health, their use in agriculture is still a relatively new concept, leaving significant room for future research and addressing many existing knowledge gaps. Future advances in agrobiotechnology involve exploring novel osmoprotectant compounds beyond those traditionally studied, while a deeper investigation of the complex interactions between soil ions and microorganisms is essential to better understand the impact of mineral fertilizers on soil salinity and osmotic stress. Optimizing application methods of osmoprotectants, including seed priming and direct application to plants, is crucial for maximizing their protective effects in salinized soils. Exploring the role of osmoprotectants in altering biofilm permeability could provide new strategies for controlling plant pathogens and improving crop health. The use of halophytes as forecrops, in combination with their rhizosphere-associated microbiomes, offers another promising approach for enhancing soil health and agricultural productivity. Furthermore, technological innovations, particularly in the stabilization and long-term storage of osmoprotectants, will be critical for their successful integration into sustainable agricultural practices.

### Unlocking potential: discovering novel osmoprotectants

Traditionally, research on microorganisms has concentrated on osmoprotectants such as proline, GB, and trehalose. However, there is still an existing gap in exploring osmoprotectants that are well-documented in plants or animals, but have not been extensively studied in microorganisms, despite evidence of their production by these organisms. Polyamines like spermidine and spermine, which are recognized for their role in stress tolerance in plants and animals (Yoon et al. 2000, Kido et al. 2019, Hasan et al. 2021) have yet to be thoroughly examined in microbial systems. These compounds are known to stabilize DNA, and proteins, and mitigate oxidative damage, underscoring their importance in cellular protection (Park and Kim 2011). Interestingly, some microorganisms also produce spermidine, suggesting its potential role in stress protection, a subject that merits further investigation (Lee et al. 2013). For example, a recent study demonstrated that the coexpression of endogenous spermidine synthase and a butanol dehydrogenase in *Clostridium thermocellum* significantly increased resistance to acetic acid and furans, enhanced ethanol production, and improved thermotolerance (Kim et al. 2023).

### Deepening the investigation of mineral fertilizers impact on soil microbiology

Excessive fertilizer application results in accumulation of nitrates, phosphates, and sulfates, leading to increased soil salinity and imposing osmotic stress on soil microbiota. This highlights the urgent need for research focused on understanding the specific impacts of these ions on soil microorganisms and overall ecosystem health. The current literature lacks comprehensive studies on how various ions influence bacterial metabolism and the mechanisms by which bacteria respond to these stresses, underscoring a critical knowledge gap regarding the full consequences of improper use of fertilizers. Rath et al. (2016) observed that different concentrations of salts, including NaCl, KCl, Na_2_SO_4_, and K_2_SO_4_, significantly affect bacterial growth. Their study found that Na_2_SO_4_, even at the lowest molar concentration (1.19 μmol salt/g soil), exhibited the highest toxicity, reducing the bacterial population up to IC50. KCl, at 1.43 μmol salt/g soil, was the next most toxic, followed by K_2_SO_4_ at 1.68 μmol salt/g soil, with NaCl being the least toxic at 1.80 μmol salt/g soil. In addition, high levels of nitrate can have significant metabolic effects on bacteria, influencing their growth. For instance, a study on *Desulfovibrio vulgaris*, a sulfate-reducing bacterium, revealed that elevated nitrate levels induced osmotic stress, as evidenced by the upregulation of genes associated with GB transport, highlighting the critical role of osmoprotectants in alleviating nitrate-induced stress (He et al. 2010a).

Fertilizer supplementation introduces not only various anions but also cations like K^+^, Na^+^, Mg^2+^, and Ca^2+^. As shown by Macêdo et al. (2019), bacterial responses to these cations can vary significantly and potential antagonistic interactions between different ions and bacteria response systems may lead to more severe toxic effects on bacteria than the concentration of individual ions alone would suggest. The toxicity of researched salts was in the following order NaCl > MgCl_2_ > CaCl_2_ and the antagonism between MgCl_2_ and NaCl was the most significant among them. Given that only a small subset of ions was examined, further research into the metabolic responses and interactions of soil bacteria with a broader range of ions is crucial. This will provide deeper insights into the multifaceted impacts of fertilizer use on microbial communities and soil health.

### Broadening knowledge about salinization effect on soil environment

A significant knowledge gap remains regarding the interaction of osmoprotectants with soil biological, chemical, and physical parameters. Research on this topic is often fragmented, with studies focusing on one condition without accounting for the broader complexity of the soil matrix. Given the diversity among soil types, comparing results across different studies is challenging and mostly ineffective, leaving a limited understanding of the real effect of supplementation of osmoprotectants and the determination of optimal doses. Furthermore, little is known about how osmoprotectants influence the mobility and availability of soil nutrients compared to soil without supplementation. Osmoprotectants like GB and proline have been shown to enhance the performance of phosphate-solubilizing bacteria under high salinity. The improved bacterial function increases the availability of phosphorus, a vital nutrient for plants. In *Bacillus* species, the presence of osmoprotectants helped maintain phosphate solubilization efficiency even under saline conditions, illustrating their potential role in improving nutrient availability in challenging soil conditions (Cherif-Silini et al. 2013). The effect of osmoprotectants on soil bacterial communities is an important area of research, particularly for enhancing bacterial resilience under stress conditions. As discussed in previous sections, osmoprotectants generally promote microbial activity and growth. However, their effect on the microbial community structure remains and metabolic functionality uncertain and warrants further investigation.

### Effect of osmoprotectants on biofilm permeability

There is considerable potential for further research into bacterial biofilms and the effect of osmoprotectants on their production under osmotic conditions. While it is established that osmoprotectants can increase biofilm production (Kapfhammer et al. 2005), their ability to enhance antibiotic permeability within biofilms presents an intriguing avenue for more effective treatments compared to antibiotics alone. For instance, osmoprotectants like acetylcholine, a GB analogue, have been shown to increase biofilm permeability to antibiotics in *P. aeruginosa*, enhancing antibiotic efficacy (Mi et al. 2014). In *Vibrio cholerae*, biofilm production increases significantly when grown in conditions mimicking its natural environment, as compared to higher salinity conditions. Notably, the expression of genes regulating osmoprotectants synthesis and transport is repressed when genes regulating EPS production are active (Shikuma et al. 2013). These findings underscore the complex interplay between osmoprotectants and biofilm formation, highlighting the need for further studies on these mechanisms before the supplementation of osmoprotectants to environmental contexts. Further research into the specific effects of osmoprotectants on bacterial communities, new strategies for soil hygienization or the targeted eradication of plant pathogens could also emerge.

### Plants growth promoting bacteria

The use of PGPB offers promising solutions for boosting plant growth and resilience in response to environmental stressors by facilitating the production of various osmoprotectants, but several efforts are still required to optimize these strategies for widespread agricultural use. These challenges include enhancing the effectiveness of PGPB, refining osmoprotectant application methods, understanding genetic and biochemical pathway regulation, and ensuring the economic viability of these approaches. A key challenge for the future is to enhance the effectiveness of PGPB by using a mixture of different bacterial strains specifically created for the plant species and the type of abiotic stress they face. Such tailored bacterial consortia can provide a broader range of benefits, enhancing targeted crop productivity and stress resilience, and optimizing microbial interactions with plants for more robust growth and adaptation under diverse environmental conditions. While previous experiments have explored this concept (Jha et al. 2011), they have not been conducted on a large scale. Thus, future research should explore the opportunity for large-scale application, potentially leading to more effective and widespread use of these tailored microbial solutions. Moreover, the impact of introducing exogenous bacterial strains or osmoprotectants on native soil microbiota must be carefully considered, as such interventions could disrupt existing microbial communities and alter soil structure and chemical properties. Future strategies should therefore be preceded by comprehensive assessments of microbiome dynamics and soil health to ensure ecological compatibility and sustainability.

### Halophytes as forecrops for salinized soils and potential source of PGPB

The utilization of halophytes as forecrops for salinized soils presents a promising strategy to improve soil health and boost subsequent crop yields. Studies indicate that halophytes can effectively remediate saline soils through various mechanisms, enhancing the soil’s physicochemical properties and microbial diversity. For instance, monocropping *Halogeton glomeratus* was found to significantly reduce soil salinity and improve soil organic matter content in Northwestern China, outperforming intercropping with *Suaeda glauca* in terms of remediation capabilities (Wang et al. 2024). Additionally, the microbiomes associated with halophytes like *Halomonas* and *Kushneria* have been identified as potential inocula that could impart salt tolerance to other crops, suggesting a pathway to enhance agricultural productivity in saline environments (Meinzer et al. 2023). Moreover, sustainable agricultural practices integrating halophytes, microbial inoculants, and soil amendments have shown potential in Mediterranean regions, further supporting the viability of halophytes in managing saline soils (Navarro-Torre et al. 2023).

Rhizosphere microorganisms, including bacteria and fungi associated with halophytes, have evolved mechanisms to tolerate and thrive in high-salinity environments, making them valuable for soil improvement. Research indicates that these microorganisms can enhance plant growth through several mechanisms: improving nutrient availability, producing phytohormones, and increasing soil water content (Sáenz-Mata et al. 2016). Kearl et al. (2019) observed increased growth and yield in alfalfa plants inoculated with bacteria isolated from the rhizospheres of three different halophyte species: *Salicornia rubra, Sarcocornia utahensis*, and *Allenrolfea occidentalis*. Plants grown in salinized soil inoculated with these microorganisms exhibited roots 2.5 times longer and fresh root biomass over 4.5 times greater than the control. Additionally, the shoot fresh weight increased by up to 21% compared to the control. These findings suggest that inoculating crops with salt-tolerant PGPR (isolated from halophytes) can significantly enhance crop yield and stress tolerance under saline conditions.

### Seed priming

Seed priming with osmoprotectants like proline and GB is a widely recognized strategy for enhancing plant resilience to abiotic stresses. For example, in *Vigna radiata*, priming seeds with GB at 200 ppm significantly improved drought tolerance, resulting in higher germination rates, enhanced seedling vigor, and increased stress tolerance indices (Saha et al. 2022). Similarly, proline was shown to enhance drought resistance in crops like wheat and maize (*Z. Mays* L.)(Dikilitas et al. 2020). The benefits of seed priming extend to various crops and environmental conditions, especially in arid and semiarid regions, where water scarcity and drought are common. Moreover, GB and proline have also been shown to improve cold tolerance in crops like rice and enhance performance under salt stress conditions (Dikilitas et al. 2020). Recent research indicates that seed priming not only improves immediate stress resilience but may also provide long-term benefits by activating defense mechanisms and enhancing overall plant health. The stress memory retained through seed priming allows for quicker activation of protective responses when plants are reexposed to similar stresses (Marthandan et al. 2020, Saha et al. 2022). This makes seed priming with osmoprotectants a promising and sustainable method for improving crop resilience to environmental stressors.

### Effective osmoprotectants supplementation to plants

As detailed in section "Prospects for osmoprotectants in agrobiotechnology", while applying osmoprotectants directly to plants has proven effective, developing cost-effective and sustainable methods for large-scale agriculture still remains a significant challenge. Advancing our understanding of the genetic and biochemical mechanisms of osmoprotectants functions in plants can lead to more targeted and efficient stress-management strategies. For instance, research into the gene transfer related to trehalose biosynthesis pathways has shown potential in enhancing drought resistance in crops like rice (Djilianov et al. 2005). Such insights can facilitate the development of transgenic plants that produce higher levels of osmoprotectants, reducing the need for external applications.

Foliar and soil applications of osmoprotectants require precise dosing to avoid phytotoxicity or ensure adequate coverage. For example, when GB is applied foliarly, it accumulates in meristematic tissues and primarily localizes to the cytosol of leaves, enhancing PSII activity and catalase (CAT) activity under stress conditions (Annunziata et al. 2019). However, the transport of osmoprotectants to key areas such as chloroplasts remains inefficient. This highlights the need for further research to optimize application methods and better understand the intracellular transport mechanisms of these solutes. The leaf age and physiological traits must also be taken into account, as they significantly influence the uptake and effectiveness of osmoprotectants. Indeed, younger leaves are generally more sensitive to environmental inputs, but are better protected against oxidative stress due to their high plasticity and lower stomatal conductance.

The understanding of osmoprotectants transport and metabolism in plants remains a significant challenge, with proline metabolism and transport serving as a prominent example. The presence of proline in phloem and xylem sap indicates its role in long-distance transport and stress response regulation, but the precise mechanisms of this transport remain underexplored (Weibull et al. 1990, Bialczyk et al. 2004).

### Application and technological aspects

The stabilization and long-term storage of osmoprotectants are critical for preserving their efficacy, particularly in pharmaceutical and agricultural applications. The methods for extending the shelf-life of these compounds can vary due to differences in their chemical structure. For example, a study on betaine capsules formulated for treating homocystinuria found that these capsules remained stable for a year when stored in airtight containers at room temperature, but their shelf-life significantly decreased in nonairtight containers at temperatures below 30°C (Hossain et al. 2013). The stability of betaine is partly due to its chemical structure, which contributes to its stability under nonenzymatic conditions (Guillory 2007). In contrast, research on extending the shelf-life and preventing degradation of other osmoprotectants, such as trehalose, is limited. Trehalose is commonly used to mitigate damage caused by storage conditions, such as low temperatures and freezing. A study on biomedical cryopreservation has demonstrated that trehalose is a nontoxic cryoprotective agent for organic compounds (Hu et al. 2023). Moreover, trehalose is proposed as a stabilizer for proteins because of its ability to bind with proteins and form a conformationally restricted and unfolded state, reducing misfolding and thereby enhancing stability (Olgenblum et al. 2021).

To develop effective products for farmland application, achieving higher concentrations of osmoprotectants solutions is essential, which would enable their use in plant watering and field irrigation. For betaine, two main strategies exist for obtaining more concentrated solutions. The first is high-performance liquid chromatography (HPLC), which involves several steps: extraction with methanolic KOH solution, purification with activated charcoal and cation exchange resin, and final separation using HPLC. This method is highly accurate and effective (Chendrimada et al. 2002). However, it is cost-intensive, making it less practical for large-scale production. Another possible method implies the use of hydrophilic interaction liquid chromatography. In this method, betaine-containing solutions are purified using strong cation exchange resins, followed by elution with acetonitrile mixed with ammonium formate buffer (in a 3:1 ratio and pH 3) (Rivoira et al. 2017). Although the second method is easier to implement, the resulting concentrated solution is unsuitable for environmental use and requires additional treatments to remove acetonitrile and adjust the low pH. Therefore, it is crucial to develop new methods that are both environmentally and economically friendly for this purpose.

## Summary

Osmoprotectants hold significant promise for agriculture, particularly in improving plant resilience and soil health in dry and saline lands. With climate change and excessive land use driving increased soil salinity, and stricter environmental regulations limiting the use of chemical inputs, osmoprotectants may offer a sustainable alternative to conventional farming practices. Their ability to alleviate osmotic stress, promote beneficial microbial activity, and enhance crop performance positions them as a valuable asset in modern farming. However, several challenges must be overcome before these compounds can be widely commercialized. Key areas include optimizing application methods for cost-effective use, scaling-up production processes, and understanding their long-term impact on soil ecosystems and transport in plants. Furthermore, refining delivery mechanisms, such as seed priming, direct plant application, and soil amendments will be crucial for maximizing their effectiveness. As the agricultural sector seeks innovative solutions to combat soil salinization and reduce dependency on chemical inputs, osmoprotectants have the potential to become a cornerstone of sustainable crop production and soil management.
